# Sexual Dimorphism of Tarsal Attachment Devices and Their Relation to Mating in Coccinellidae

**DOI:** 10.1002/jmor.70041

**Published:** 2025-04-03

**Authors:** Valerio Saitta, Manuela Rebora, Silvana Piersanti, Giorgia Carboni Marri, Paolo Masini, Elena Gorb, Alessia Iacovone, Gianandrea Salerno, Stanislav Gorb

**Affiliations:** ^1^ Dipartimento di Scienze Agrarie, Alimentari e Ambientali University of Perugia Perugia Italy; ^2^ Dipartimento di Scienze Agrarie e Forestali University of Palermo Palermo Italy; ^3^ Dipartimento di Chimica, Biologia e Biotecnologie University of Perugia Perugia Italy; ^4^ Department of Functional Morphology and Biomechanics Zoological Institute, Kiel University Kiel Germany; ^5^ CBC Bioplanet Cesena Italy

**Keywords:** beetles, coevolution, Coleoptera, disco‐setae, hairy pads, mating behavior

## Abstract

This study investigates the coevolution of male attachment devices and female elytral morphology in coccinellid beetles, focusing on the sexual dimorphism of claws and adhesive pads. We analyzed 11 species from different tribes with different feeding regime, examining the structure of male and female attachment organs (claws and hairy pads) in relation to the surface structure of female elytra. Our findings show that disco‐setae, which enhance adhesion during mating, are present only in males of some species and are localized on the hairy pads of their legs. These setae exhibit morphological adaptations based on the surface structure of female elytra, with larger discoid setal tips in species with smooth elytra and smaller tips in those with hairy elytra. Additionally, male beetles with hairy elytra possess dimorphic claws, which enhance attachment efficiency compared to species with smooth elytra, where claw dimorphism is less pronounced. Our results reveal that sexual dimorphism in hairy pads is more pronounced in larger species, where claw dimorphism is absent, while in smaller species, claw dimorphism alone suffices for effective attachment. These findings contribute to a deeper understanding of the evolutionary dynamics shaping attachment adaptations in Coccinellidae, with implications for reproductive strategies, pest management, and ecological interactions in this diverse beetle family.

## Graphical abstract

We explore the coevolution of male attachment devices and female elytral morphology in coccinellids. Specifically: (1) Disco‐setae are present in males of some species, located on the hairy pad which during mating hold on female elytra. (2) The area of male disco‐setae is extended when females have smooth elytra, and reduced when females have hairy elytra. (3) Males with hairy elytra possess bifid claws. (4) Sexual dimorphism in pad setae evolved only in larger species, where claw dimorphism is absent.

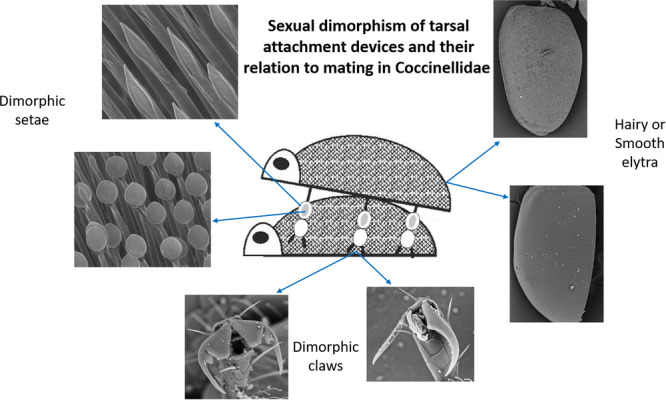

## 1 Introduction

Insects, the most diverse taxon in the animal kingdom (Wiegmann and Trautwein 2014), are characterized by complex chemical and mechanical interactions with their environment (Ryan and Byrne 1988; Frazier and Chyb 1995) and diverse adaptations. Among these adaptations, insects exhibit an excellent ability to attach to surfaces, primarily due to specialized attachment devices generally located on tarsi, which include sclerotized claws and soft adhesive pads (Gorb 2001). Claws are used to interlock with coarsely rough surfaces (Dai et al. 2002) or plants trichomes (Saitta et al. 2022; Salerno, Rebora, Piersanti, Saitta et al. 2022) and their different shapes are often associated with specific substrates, where insects live (Friedemann et al. 2014; Salerno, Rebora, Piersanti, Saitta et al. 2022). On the other hand, the pads can adhere to several types of substrates (Gorb 2001; Dirks and Federle 2011). The high resilin content in these pads (Michels and Gorb 2012; Peisker et al. 2013; Rebora et al. 2018, 2021) make them soft and flexible, allowing for maximum contact with smooth and microrough surfaces (Beutel and Gorb 2001; Gorb 2001). Two types of pads can be distinguished: (1) hairy pads, covered with relatively long and flexible setae, which are commonly found in flies, beetles, and earwigs, and (2) smooth pads, such as arolia, typically observed in cockroaches and bees or euplantulae observed in grasshoppers, stick insects and mantids. The material flexibility in both types of the pads allows them to adapt and optimize contact with a variety of substrates (reviewed in Gorb 2001, 2005, 2008; Federle 2004). In all insect species, the interaction between adhesive organs and the substrate is further facilitated by a thin layer of liquid pad secretion, which enhances the contact area, particularly on uneven surfaces, and generates capillary forces (see review by Dirks and Federle 2011).

Sexual dimorphism in attachment ability and structure of adhesive devices has been observed in various insect species, often linked to the need to adhere to the host surface during oviposition as observed in hymenopteran parasitoids (Rebora et al. 2022; Salerno et al. 2024) or to the female during mating, such as the case in some Coleoptera (Gorb et al. 2010; Heepe et al. 2017; Voigt et al. 2008, 2017). In many species of beetles, there are distinct differences between shapes of the adhesive setae in females and males (Stork 1980; Voigt et al. 2008). Females typically possess needle‐shaped or slightly spatulated setae, while males have, in addition to these, highly specialized setae with a round to oval plate at the distal end of a rigid shaft, called disco‐setae (terminology according to Stork 1980). To the best of our knowledge, the disco‐setae are present in more than 85 Coleoptera species (in both the Adephaga and Polyphaga suborders) belonging to Caraboidea, Staphylinoidea, Coccinellidae, Chrysomeloidea, and Curculionoidea (Liu and Liang 2016; Stork 1980; Voigt et al. 2008). Data concerning the presence of disco‐setae for numerous families of Coleoptera are lacking. The sexual dimorphism is spectacular in Dytiscinae (Bergsten et al. 2001). These specialized setae are able to provide strong adhesive performance on smooth surfaces (Gorb, Varenberg et al. 2007), such as female elytra during mating (Alcock 2006). In line with this, higher adhesive forces have been measured in males on smooth surfaces compared to females in *Harmonia axyridis* Pallas (Coleoptera: Coccinellidae) (Gorb et al. 2019), probably due to the sexual dimorphism in the microstructure of their adhesive pads. A similar difference in attachment ability to smooth surfaces between males and females has also been observed in the rosemary beetles *Chrysolina americana* Linneaus (Coleoptera: Chrysomelidae), where pull‐off force measurements have been performed on both female elytra and flat glass (Voigt et al. 2017). It has been found that on convex elytra, insects performed better than on flat glass, with males exerting higher force than females.

Given the necessity for male beetles to firmly adhere on female elytra during mating, males of different species have evolved different copulation modalities in relation to the elytral morphology. In general, female elytra provide support for males during mating (Goczał and Beutel 2023) and their surfaces in Coleoptera can be either smooth (Heepe et al. 2017), hairy due to microtrichia coverage (Gorb and Gorb 2020), exhibit reticulated patterns or conspicuous longitudinal furrows as seen in dytiscids (Drotz et al. 2010). In this context, sexually antagonistic coevolution has been suggested in male and female diving beetles (family Dytiscidae), where the reticulated structure of the female's elytra may reduce males adhesion during copulation (Karlsson Green et al. 2013). In some species of Coleoptera, males hold the female's abdomen with all six legs, as observed in *Gastrophysa viridula* De Geer (Coleoptera: Chrysomelidae) (Matsumura et al. 2023). In other beetles, such as *C. americana* (Linneaus), the hindlegs may touch the substrate (Voigt et al. 2017), while in *Leptinotarsa decemlineata* Say (Coleoptera: Chrysomelidae), the fore‐ and midlegs are positioned on elytra and the hindlegs are wedged on the elytra margin and the abdominal sclerites (Voigt et al. 2008).

The family Coccinellidae includes more than 6000 described species (Ahmad et al. 2024; Canepari 2009), exhibiting a wide trophic range that includes myrmecophages (Vantaux et al. 2010), micophages (Younes et al. 2015), phytophages (Piersanti et al. 2022) causing serious damages to crops, and zoophages (Heepe et al. 2017) employed in biological control programs (Hodek et al. 2012). The interactions between coccinellids and their environment have been extensively studied, particularly regarding their chemical and mechanical ecology (Hodek 1973; Gordon 1985; Iperti and Prudent 1986; Roy and Wajnberg 2008; Pell et al. 2008; Gorb et al. 2008, 2017, 2019; Gorb and Gorb 2020; Hodek et al. 2012; Moon et al. 2012; Yao et al. 2021; Salerno, Rebora, Piersanti, Büscher et al. 2022; Salerno, Rebora, Piersanti, Saitta et al. 2022; Piersanti et al. 2022, 2023; Saitta et al. 2023, 2025; Sevarika and Romani 2024). However, only limited information is available about the male attachment ability and behavior during copulation in Coccinellidae.

In this study, we hypothesize a coevolution between the morphology of claws and adhesive setae of males and the surface structure of female elytra. Using scanning electron microscopy, we examined 11 species of Coccinellidae to assess sexual dimorphism in the structure of attachment devices (claws and hairy pads) of males and females in relation with the elytral surface. Additionally, behavioral observations during mating were performed in order to determine, which legs and attachment devices are used by males to adhere to females. Our data aim to provide insights on the evolutionary dynamics shaping the diversity of tarsal attachment devices and their role in reproductive strategies within this ecologically important beetle family.

## 2 Materials and Methods

### 2.1 Examined Species

For this study, we selected 11 species of Coccinellidae with different feeding habits (from zoophagous to phytophagous) (Table S1) spread over the clade (see Che et al. 2021). In particular, we selected *H. axyridis*, *Propylea quatuordecimpunctata* (L.), *Coccinella septempunctata* (L.), and *Adalia bipunctata* (L.) from the tribe Coccinellini, *Chnootriba elaterii* (Rossi), *Henosepilachna argus* Geoffroy, and *Subcoccinella vigintiquatuorpunctata* L. from the tribe Epilachnini, *Nephus conjunctus* Wollaston and *Cryptolaemus montrouzieri* (Muslant) from the tribe Scymnini, *Exochomus quadripustulatus* (L.) from the tribe Chilocorini, and *Delphastus catalinae* (Horn) from the tribe Seranginini.

*H. axyridis*, *C. septempunctata*, *C. elaterii, H. argus*, and *S. vigintiquatuorpunctata* were reared in net cages (300 mm × 300 mm × 300 mm) (Vermandel, Hulst, The Netherlands) under controlled conditions (16:8 D:L photoperiod, temperature of 25 ± 1°C, and a relative humidity of 60 ± 10%) in the Department of Agricultural, Food and Environmental Sciences, University of Perugia, Italy. *H. axyridis* and *C. septempunctata* were reared on *Vicia faba* L. (Fabaceae) plants infested with *Aphis fabae* Scopoli (Hemiptera: Aphididae), *C. elaterii*—on *Cucumis melo* L. (Cucurbitaceae), *H. argus*—on *Ecballium elaterium* (L.) (Cucurbitaceae), and *S. vigintiquatuorpunctata* on *Silene alba* (Mill) (Caryophyllaceae). Adults of both sexes of *P. quatuordecimpunctata*, *A. bipunctata*, *N. conjunctus*, *C. montrouzieri*, *E*. *quadripustulatus*, and *D. catalinae* were kindly supplied by the company (Bioplanet, Cesena, Italy).

### 2.2 Scanning Electron Microscopy (SEM) and Image Analysis

Legs and elytra of both sexes were cut off from animals anesthetized by CO_2_, then dehydrated, mounted on metal stubs using double‐sided adhesive carbon tape, sputter‐coated with gold–palladium (thickness 8 nm), and observed in a Hitachi Tabletop SEM TM‐3000 (Hitachi High‐Technologies Corp., Tokyo, Japan) at 15 kV. In both sexes, the claws, the ventral side of the proximal and distal tarsomere of the fore‐, mid‐ and hindlegs, and the elytra were observed.

**Figure 1:**
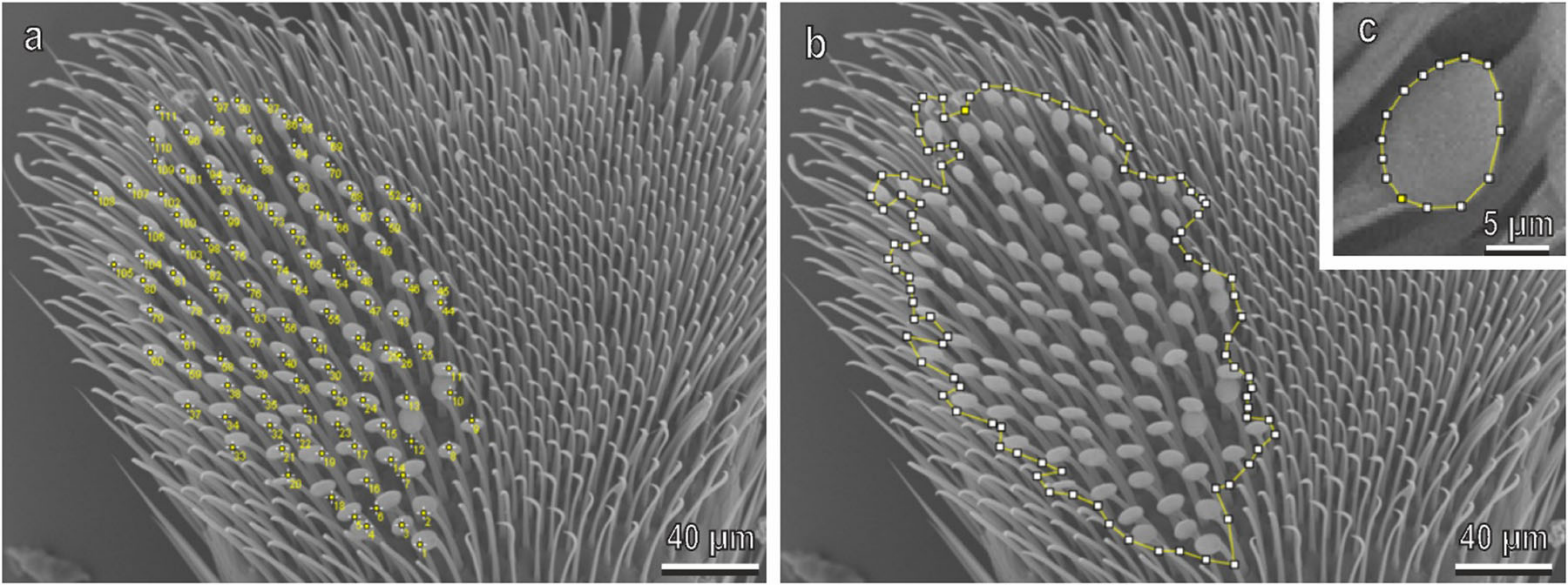
The distal tarsomere of the male midleg *in Harmonia axyridis*: (a) disco‐setae; (b) area covered by disco‐setae; and (c) area of a discoidal terminal plate of a disco‐seta.

The obtained images were analyzed using the image analysis program ImageJ (Schneider et al. 2012). In our analysis, we considered the “male setae,” that is, disco‐setae, found only in males and used for holding females during copulation according to Stork (1980). In particular, we calculated:

– density of the disco‐setae (Figure 1a,b), that is, number of disco‐setae on area covered by these setae, on the distal and proximal tarsomeres of for‐, mid‐, and hindlegs (*n* = 6);
– percentage of the area covered by disco‐setae in relation to the total area of the tarsomere for the distal and proximal tarsomere of for‐, mid‐, and hindlegs (*n* = 6);
– area of the discoidal terminal plate of the disco‐setae (*n* = 6) in the proximal tarsomere of the forelegs (Figure 1c). We considered the male foreleg proximal tarsomere as a representative segment of the tarsus, because the foreleg is always positioned on female elytra. Data among the different species were compared using one‐way analysis of variance (ANOVA) followed by Tukey's test for multiple comparison.
– density of elytra setae on female elytra calculated in a square of 300 µm × 300 µm.

### 2.3 Contact Angle Measurements on Elytra

Wettability of female elytra in 11 Coccinellidae species was characterized by determining the contact angle (CA) of water using a high‐speed optical CA measuring instrument OCAH 200 (Dataphysics Instruments GmbH, Filderstadt, Germany). We used Aqua millipore and different drop methods: 1 µL droplet for *H. axyridis*, *A. bipunctata*, *C. septempunctata, P. quatuordecimpunctata*, *E*. *quadripustulatus* (sessile drop method), *C. elaterii* and *H. argus* (sessile drop needle‐in method), 0.5 µL for *C. montrouzieri* and *S. vigintiquatuorpunctata* (sessile drop needle‐in), and 0.4 µL for *N. conjunctus* (sessile drop needle‐in) and *D. catalinae* (sessile drop). Ten female elytra (*n* = 10) of each species were measured.

### 2.4 Behavioral Observations

For each of the 11 species, we observed and photographed 5–15 couples during mating and recorded the positions of the male fore‐, mid‐, and hindleg on the female body or substrate. Some species (*S. vigintiquatuorpunctata*, *N. conjunctus*, *C. montrouzieri*, *E*. *quadripustulatus*, and *D. catalinae*) were observed under the optical stereomicroscope Wild M420 (Leica Microsystems GmbH, Wetzlar, Germany) connected with Koppace FHD Camera V 2.0, while *H. axyridis*, *P. quatuordecimpunctata*, *C. septempunctata*, *A. bipunctata*, *C. elaterii*, and *H. argus* were photographed with a Nikon D90 digital camera.

## 3 Results

### 3.1 *Harmonia axyridis* (Tribe: Coccinellini)

**Figure 2:**
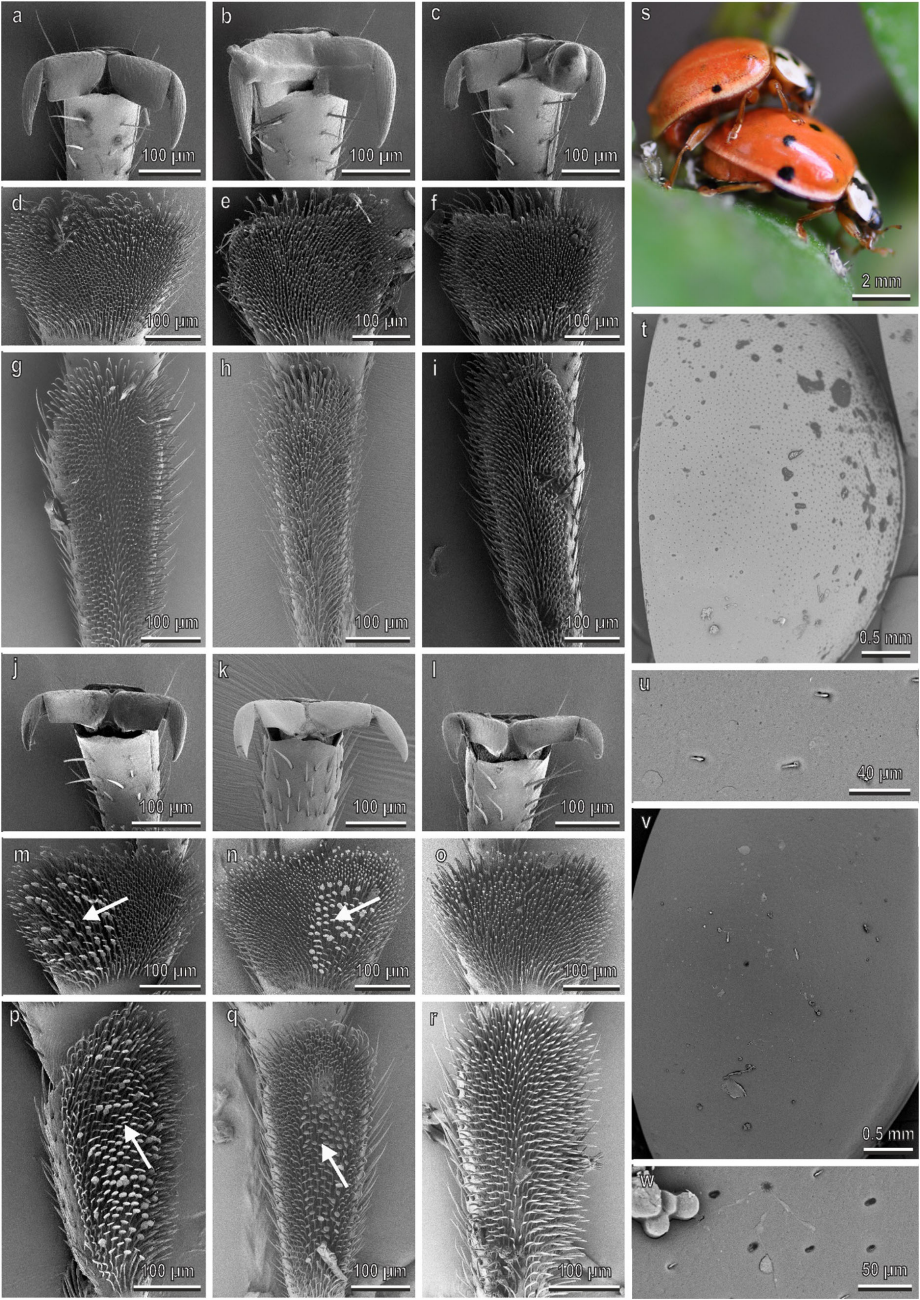
*Harmonia axyridis*. Female attachment devices (a–i), female elytra (t and u), male attachment devices (j–r), and male elytra (v and w) in the SEM: claws (a–c and j–l), distal (d–f and m–o) and proximal (g–i and p–r) tarsomeres of the fore‐ (a, d, g, j, m, p), mid‐ (b, e, h, k, n, q), and hindlegs (c, f, i, l, o, r). (s) The position of the male on the female during mating is shown. Arrows denote the disco‐setae on the male tarsi.

**Figure 3:**
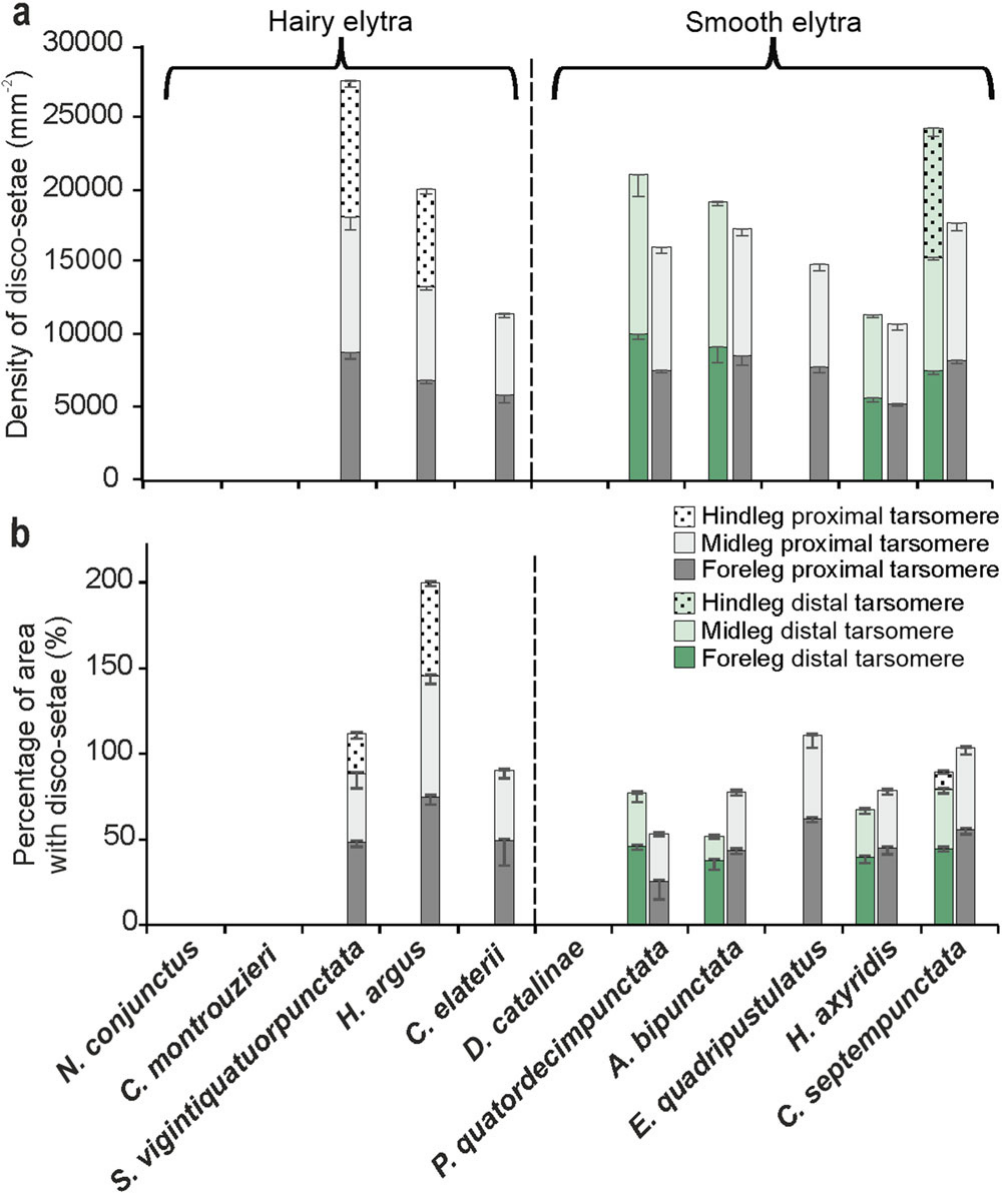
Density of disco‐setae (mean ± SE) (a) and percentage of area covered by disco‐setae (mean ± SE) (b) in the males' tarsomere in relation to the hairy or smooth females' elytra in 11 Coccinellidae species.

**Figure 4:**
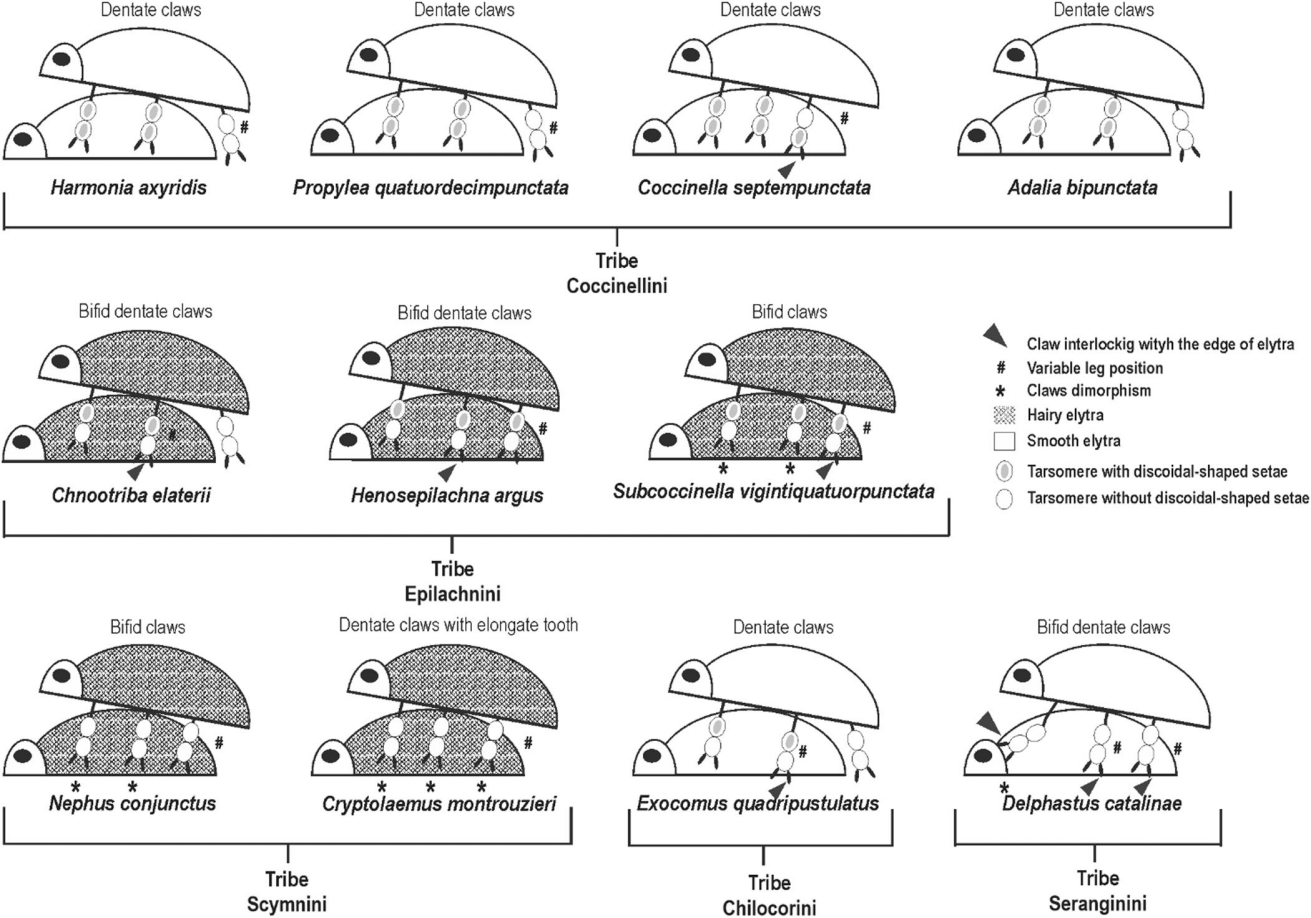
Mating postures of 11 Coccinellidae species belonging to different tribes. For males, presence or absence of dimorphic claws (asterisk), the disco‐setae (gray points) in the fore‐, mid‐, and hindlegs and their position on the females' elytra (placed or interlocked (arrowhead)) and/or on the substrate are presented in relation to the hairy or smooth females' elytra.

The pretarsal claws of *H. axyridis* are similar on the fore‐, mid‐, and hindlegs and show a deep cleft separating a wide basal tooth (dentate or appendiculate claws) from the distal portion of the claw (Figure 2a–c). No sexual dimorphism is present in the claw morphology (Figure 2a–c,j–l). The first and the second tarsomere of the fore‐, mid‐, and hindlegs have each a hairy pad with numerous tenent setae (Figure 2d–i,m–r). Only in male, disco‐setae with a wide discoidal terminal plate are visible in the first and second tarsomeres of the fore‐ and midlegs (Figure 2m,n,p,q). In the second tarsomere, the disco‐setae are concentrated in the lateral portion of the pad (Figure 2m,n). The density of the disco‐setae is similar on both tarsomeres and on both legs (Figure 3a). In the foreleg, the percentage of the area covered by disco‐setae on the total area of the tarsomere is about 40% on both tarsomeres, while in the midleg it is about 30% in both tarsomeres (Figure 3b). Elytra in both sexes are rather smooth with few scattered pits and short hairs (Figure 2t–w) and a thin layer of wax covers the elytra cuticle (Figure 2t–w). The female elytra are hydrophobic: the CA of water is 91.5° ± 0.9°. During mating, the male is contacting the female elytra with the mid‐ and forelegs. The position of the hindlegs is variable and they are often in contact with the substrate (Figures 2s and 4).

### 3.2 *Propylea quatuordecimpunctata* (Tribe: Coccinellini)

**Figure 5:**
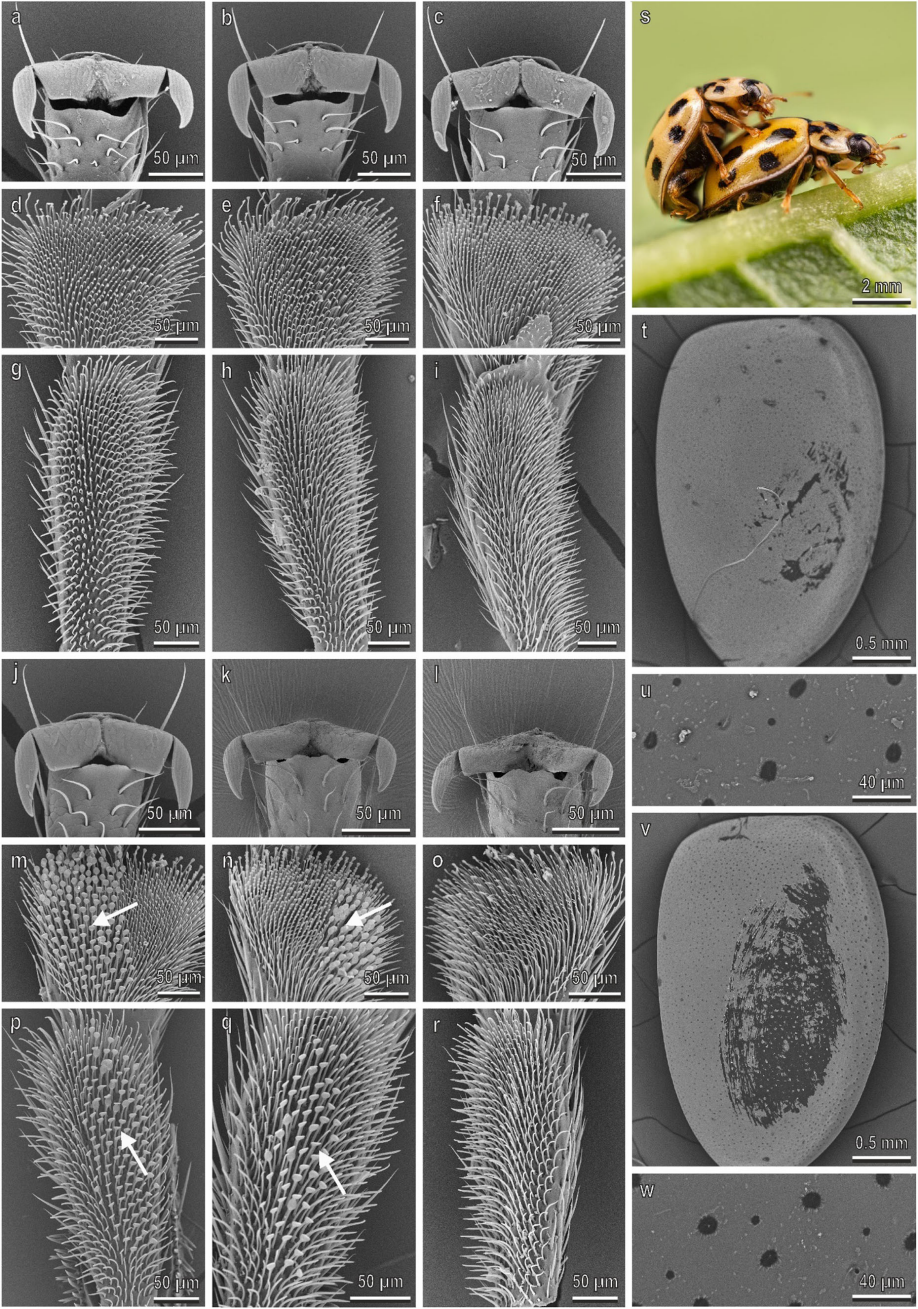
*Propylea quatuordecimpunctata*. Female attachment devices (a–i), female elytra (t and u), male attachment devices (j–r), and male elytra (v and w) in the SEM: claws (a–c and j–l), distal (d–f and m–o) and proximal (g–i and p–r) tarsomeres of the fore‐ (a, d, g, j, m, p), mid‐ (b, e, h, k, n, q), and hindlegs (c, f, i, l, o, r). (s) The position of the male on the female during mating is shown. Arrows denote the disco‐setae on the male tarsi.

In *P. quatuordecimpunctata* (Figure 5), claws, tarsi, and elytra in both sexes are very similar to those in *H. axyridis*. The density of the disco‐setae in the second tarsomere is higher than 10,000 mm^−2^ in the second tarsomere and about 8000 mm^−2^ in the first tarsomere in the fore‐ and midlegs (Figure 3a). In both legs, the percentage of the area covered by disco‐setae is about 30% in all the tarsomeres (Figure 3b). A thin wax layer covers the elytra cuticle (Figure 2t–w). The water CA with the female elytra is 88.5° ± 0.8°. During mating, the male is contacting the female similar to the way described in *H. axyridis* (Figures 4 and 5s).

### 3.3 *Coccinella septempunctata* (Tribe: Coccinellini)

**Figure 6:**
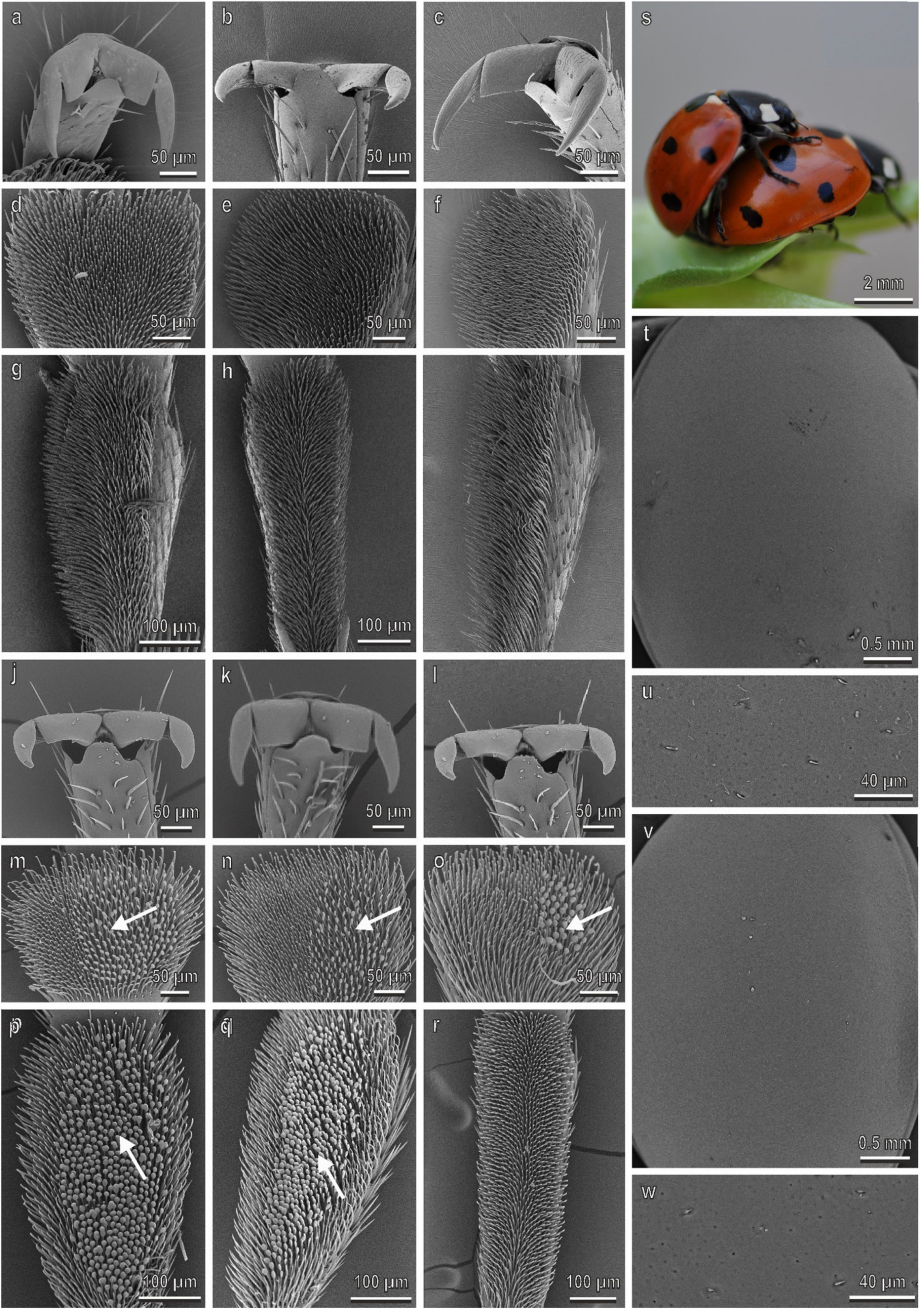
*Coccinella septempunctata*. Female attachment devices (a–i), female elytra (t and u), male attachment devices (j–r), and male elytra (v and w) in the SEM: claws (a–c and j–l), distal (d–f and m–o) and proximal (g–i and p–r) tarsomeres of the fore‐ (a, d, g, j, m, p), mid‐ (b, e, h, k, n, q), and hindlegs (c, f, i, l, o, r). (s) The position of the male on the female during mating is shown. Arrows denote the disco‐setae on the male tarsi.

In *C. septempunctata* (Figure 6), claws, tarsi and elytra in both sexes are very similar to those in *H. axyridis* with the only exception that the male disco‐setae are visible in a low number also on the second tarsomere of the hindlegs (Figure 6o). Elytra in both sexes are very smooth with some scattered pits and short hairs (Figure 6t–w). The density of the disco‐setae in the second tarsomere is about 8000 mm^−2^ in all the three legs (Figure 3a). In the first and second tarsomeres of the fore‐ and midlegs, the percentage of the area covered by disco‐setae is higher than 40%, while in the second tarsomere of the hindlegs it is about 10% (Figure 3b). A thin layer of wax covers the elytra cuticle (Figure 6t–w). The water CA with female elytra is 83.4° ± 1.9° (hydrophilic). During mating, the male is in contact with the female elytra using the mid‐ and forelegs. The position of the hindlegs is variable, often in contact with the female elytra showing the claw interlocking with the elytra edge (Figures 4 and 6s).

### 3.4 *Adalia bipunctata* (Tribe: Coccinellini)

**Figure 7:**
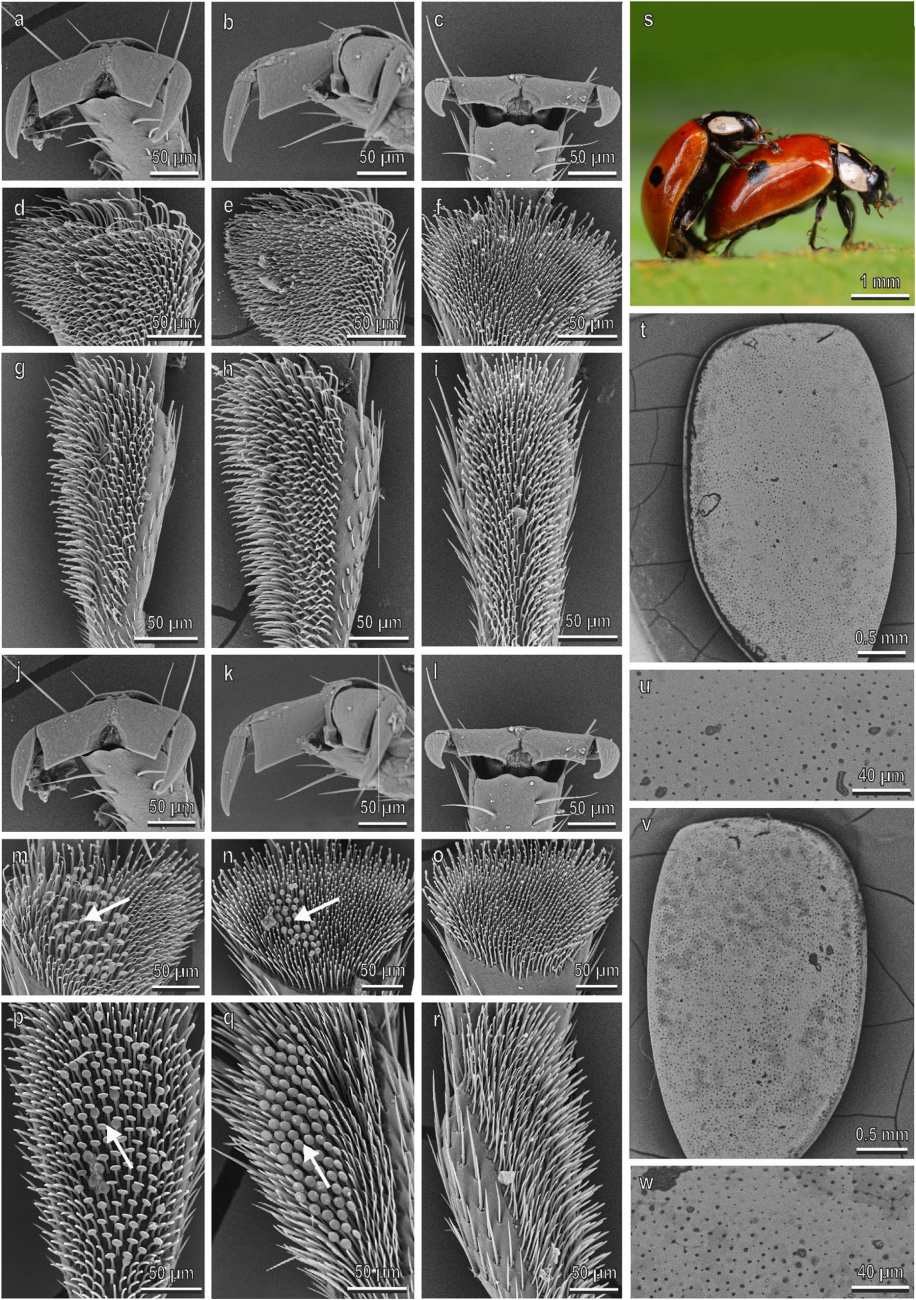
*Adalia bipunctata*. Female attachment devices (a–i), female elytra (t and u), male attachment devices (j–r), and male elytra (v and w) in the SEM: claws (a–c and j–l), distal (d–f and m–o) and proximal (g–i and p–r) tarsomeres of the fore‐ (a, d, g, j, m, p), mid‐ (b, e, h, k, n, q), and hindlegs (c, f, i, l, o, r). (s) The position of the male on the female during mating is shown. Arrows denote the disco‐setae on the male tarsi.

In *A. bipunctata* (Figure 7), claws and tarsi in both sexes are very similar to those in *H. axyridis*. The density of the disco‐setae is about 9000 mm^−2^ and is similar in the two tarsomeres and in the two legs (Figure 3a). In the foreleg, the percentage of the area covered by disco‐setae is about 40% in both tarsomeres, while in the midleg such percentage is about 34% in the first tarsomere and about 14% in the second tarsomere (Figure 3b). The elytra are rather smooth in both sexes with numerous pits; the cuticle is covered with a thin layer of wax (Figure 7t–w). The water CA with the female elytra is 90° ± 1°. During mating, male contacts the female elytra with the mid‐ and forelegs, while the hindlegs are contacting the substrate (Figures 4 and 7s).

### 3.5 *Chnootriba elaterii* (Tribe: Epilachnini)

**Figure 8:**
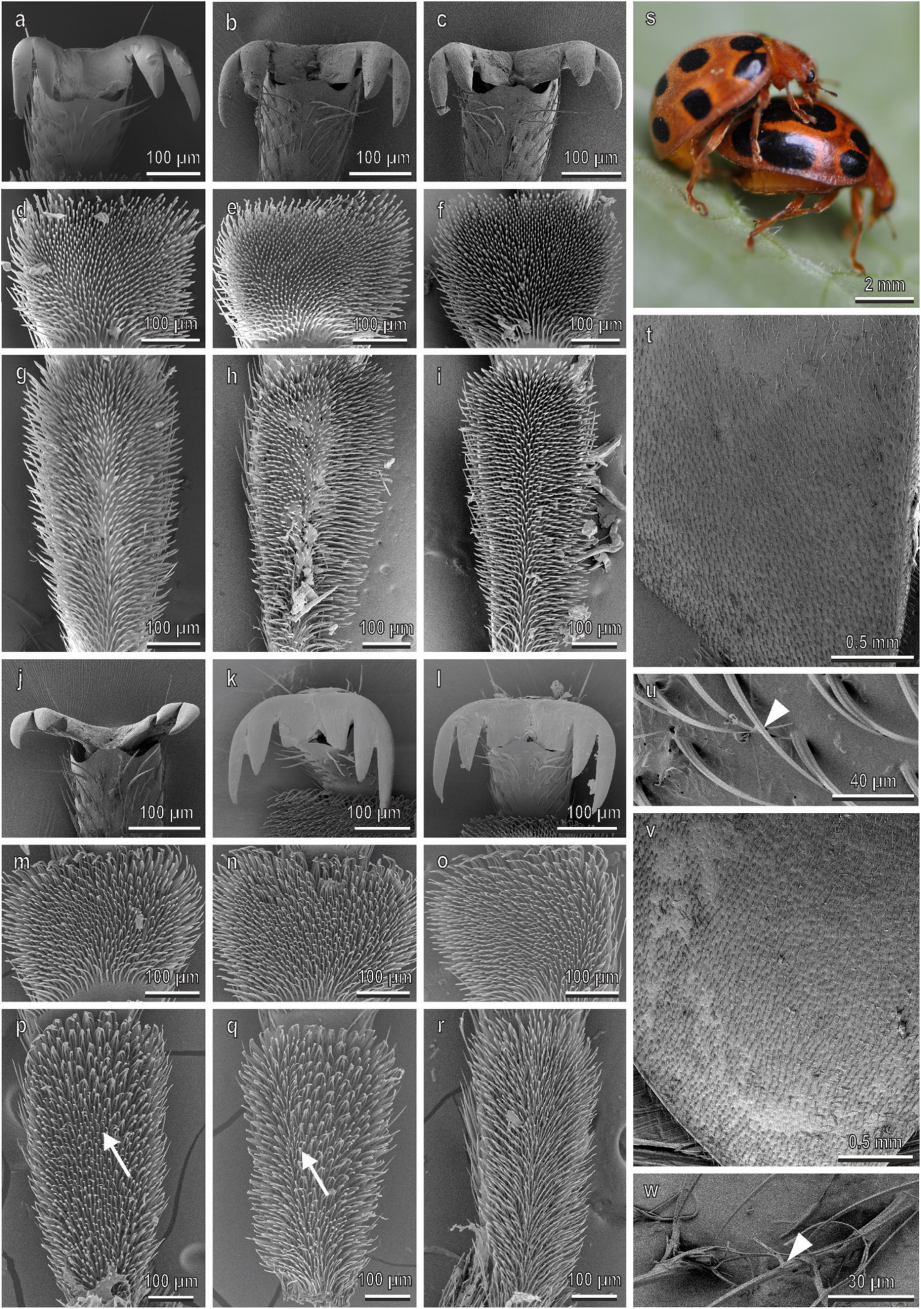
*Chnootriba elaterii*. Female attachment devices (a–i), female elytra (t and u), male attachment devices (j–r), and male elytra (v and w) in the SEM: claws (a–c and j–l), distal (d–f and m–o) and proximal (g–i and p–r) tarsomeres of the fore‐ (a, d, g, j, m, p), mid‐ (b, e, h, k, n, q), and hindlegs (c, f, i, l, o, r). (s) The position of the male on the female during mating is shown. Arrows denote the disco‐setae on the male tarsi; arrowheads point to the setae on male and female elytra.

In *C. elaterii*, the pretarsal claws are similar in all the legs (Figure 8a–c). They are bifid and show a basal tooth (bifid dentate or bifid appendiculate claws) separated from the distal bifid claw by a deep cleft. There is no sexual dimorphism in the claw morphology (Figure 8a–c,j–l). The first and second tarsal segments bear each a hairy pad with numerous tenent setae (Figure 8d–i,m–r). In males, the first tarsomere of the fore‐ and midlegs has disco‐setae with a discoidal terminal plate, which is very reduced compared with the above reported species (Figure 8p,q). The density of the disco‐setae is about 6000 mm^−2^ and is similar in both legs (Figure 3a). The percentage of the area covered by disco‐setae is about 45% in both legs (Figure 3b). Long setae cover the elytra in both sexes (Figure 8t–w); their density on the female elytra is 600 ± 103.2 mm^−2^. Among the setae, wax filaments extruded from cuticular pores are visible (Figure 2u,w). The female elytra have superhydrophobic properties showing a very high (161.3° ± 0.9°) CA of water. During mating, male keeps its forelegs in contact with the female elytra, whereas its midlegs are either contacting the female elytra or the claws are interlocking with the elytra edge; the hindlegs are on the substrate (Figures 4 and 8s).

### 3.6 *Henosepilachna argus* (Tribe: Epilachnini)

**Figure 9:**
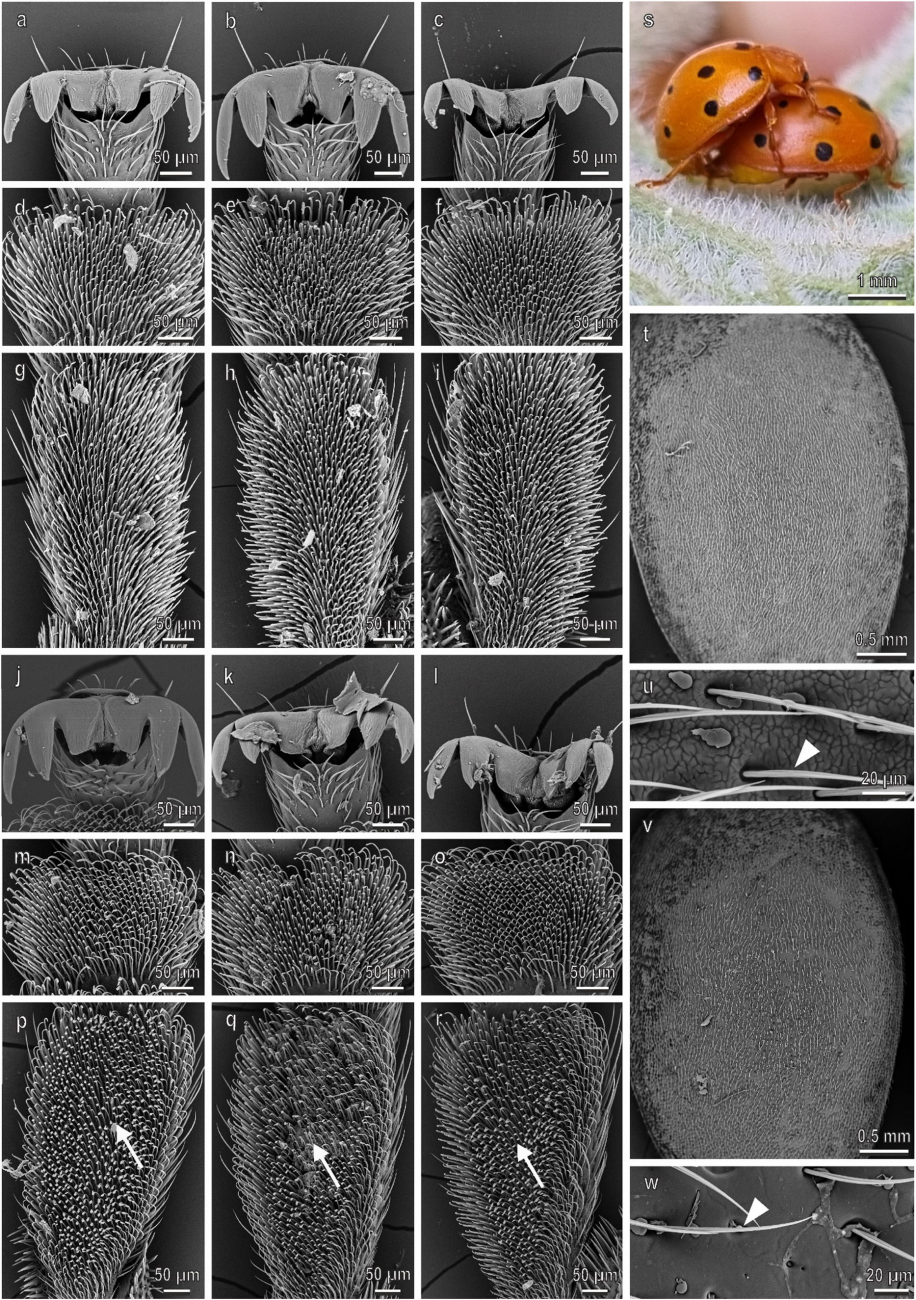
*Henosepilachna argus*. Female attachment devices (a–i), female elytra (t and u), male attachment devices (j–r), and male elytra (v and w) in the SEM: claws (a–c and j–l), distal (d–f and m–o) and proximal (g–i and p–r) tarsomeres of the fore‐ (a, d, g, j, m, p), mid‐ (b, e, h, k, n, q), and hindlegs (c, f, i, l, o, r). (s) The position of the male on the female during mating is shown. Arrows denote the disco‐setae on the male tarsi; arrowheads point to the setae on male and female elytra.

In *H. argus* (Figure 9), claws, tarsi and elytra in both sexes are very similar to those in *C. elaterii*. The male hairy pads show disco‐setae with a very reduced discoidal terminal plate only in the first tarsomere of all the three legs (Figure 9p–r). Both the density of disco‐setae (about 6500 mm^−2^) (Figure 3a) and the percentage of the area covered by disco‐setae (about 70%) (Figure 3b) are similar in the three legs. The density of the elytra setae on the female elytra is 667 ± 17 mm^−2^. An extrusion of wax is visible among the setae of the elytra (Figure 9u,w). Water CA on the female elytra is 162.1.5° ± 1.2°. During mating (Figures 4 and 9s), male keeps its forelegs in contact with the female elytra. Claws of the midlegs are interlocking with the elytra edge and the hindlegs are either on the elytra or on the substrate (or even raised).

### 3.7 *Subcoccinella vigintiquatuorpunctata* (Tribe: Epilachnini)

**Figure 10:**
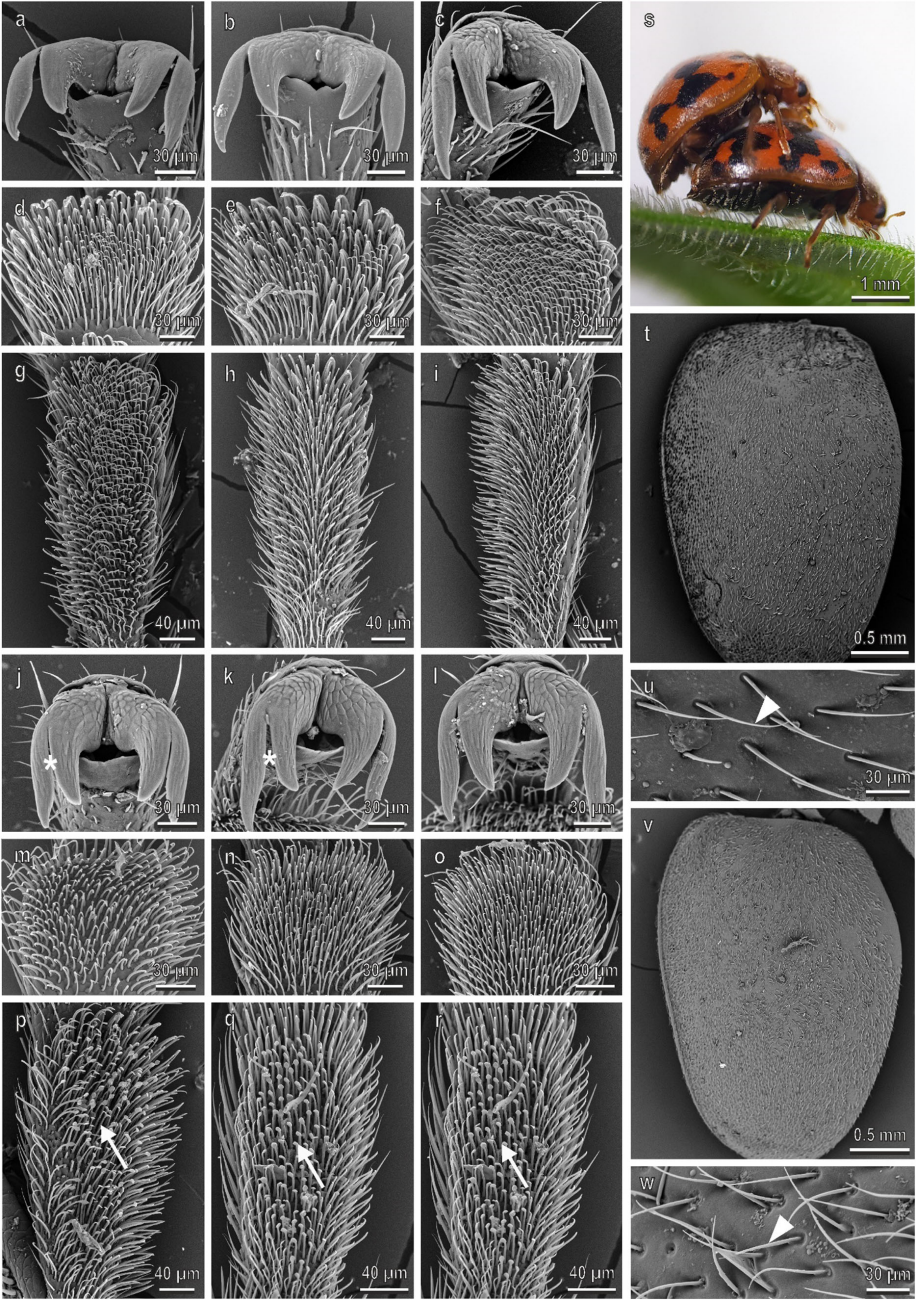
*Subcoccinella vigintiquatuorpunctata*. Female attachment devices (a–i), female elytra (t and u), male attachment devices (j–r), and male elytra (v and w) in the SEM: claws (a–c and j–l), distal (d–f and m–o) and proximal (g–i and p–r) tarsomeres of the fore‐ (a, d, g, j, m, p), mid‐ (b, e, h, k, n, q), and hindlegs (c, f, i, l, o, r). (s) The position of the male on the female during mating is shown. Arrows denote the disco‐setae on the male tarsi; arrowheads point to the setae on male and female elytra. The dimorphic claws of male are characterized by a narrower cleft (asterisk) and longer claws compared to female.

In *S. vigintiquatuorpunctata*, the pretarsal claws are bifid, (without any basal tooth) (Figure 10a–c). There is slight sexual dimorphism in the claw morphology of the forelegs and midlegs, with the male showing a narrower cleft and slightly longer claws compared with female (compare Figure 10a,b with 10j,k). In the male first tarsomere of all the legs, disco‐setae are visible (Figure 10p–r). The density of disco‐setae is about 9000 mm^−2^ and is similar in the three legs (Figure 3a). The percentage of the area covered by disco‐setae is about 40% in the foreleg and midleg and 20% in the hindleg (Figure 3b). Long setae cover the elytra in both sexes (Figure 10t–w). The density of the setae on the female elytra is 604 ± 24 mm^−2^. Among the elytra setae, the elytra surface bears wax extrusions (Figure 2u,w). The female elytra show a high (138° ± 4.8°) CA of water (highly hydrophobic). During mating (Figures 4 and 10s), male keeps its forelegs and midlegs in contact with the female elytra, whereas claws of its hindlegs are interlocking with the female elytra edge.

### 3.8 *Nephus conjunctus* (Tribe: Scymnini)

**Figure 11:**
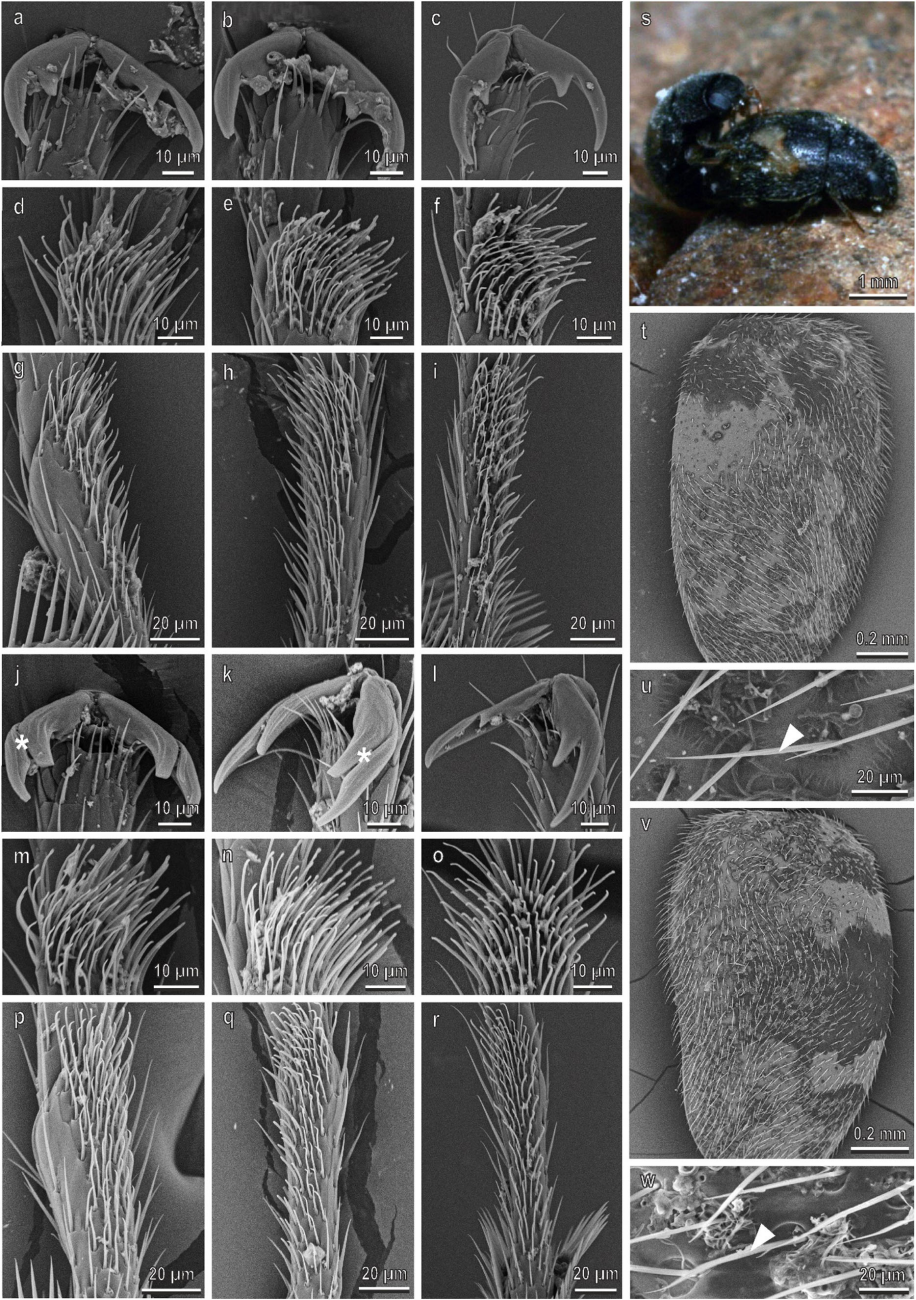
*Nephus conjunctus*. Female attachment devices (a–i), female elytra (t and u), male attachment devices (j–r), and male elytra (v and w) in the SEM: claws (a–c and j–l), distal (d–f and m–o) and proximal (g–i and p–r) tarsomeres of the fore‐ (a, d, g, j, m, p), mid‐ (b, e, h, k, n, q), and hindlegs (c, f, i, l, o, r). (s) The position of the male on the female during mating is shown. Arrowheads point to the setae on male and female elytra. The dimorphic claws of male are characterized by a narrower cleft (asterisk) and longer claws compared to female.

In *N. conjunctus*, the pretarsal claws are bifid in both sexes and, in the female, they are similar in the fore‐, mid‐, and hindlegs (Figure 11a–c). There is sexual dimorphism in the claw morphology of the fore‐ and midlegs: the male has a much narrower cleft and longer claws compared to the female (compare Figure 11a,b with 11j,k). No sexual dimorphism is present in the hairy pads, since disco‐setae are absent in the male tarsomeres (Figure 10m–r). Long setae cover the elytra in both sexes (Figure 11t–w). The density of the setae on the female elytra is 989 ± 39 mm^−2^. Wax filaments are well visible among the setae of the elytra (Figure 2u,w). The female elytra show a water CA of 166.0° ± 1.3° (superhydrophobic). During mating (Figures 4 and 11s), male has its fore‐ and midlegs in contact with the female elytra, while its hindlegs are either on the elytra or raised.

### 3.9 *Cryptolaemus montrouzieri* (Tribe: Scymnini)

**Figure 12:**
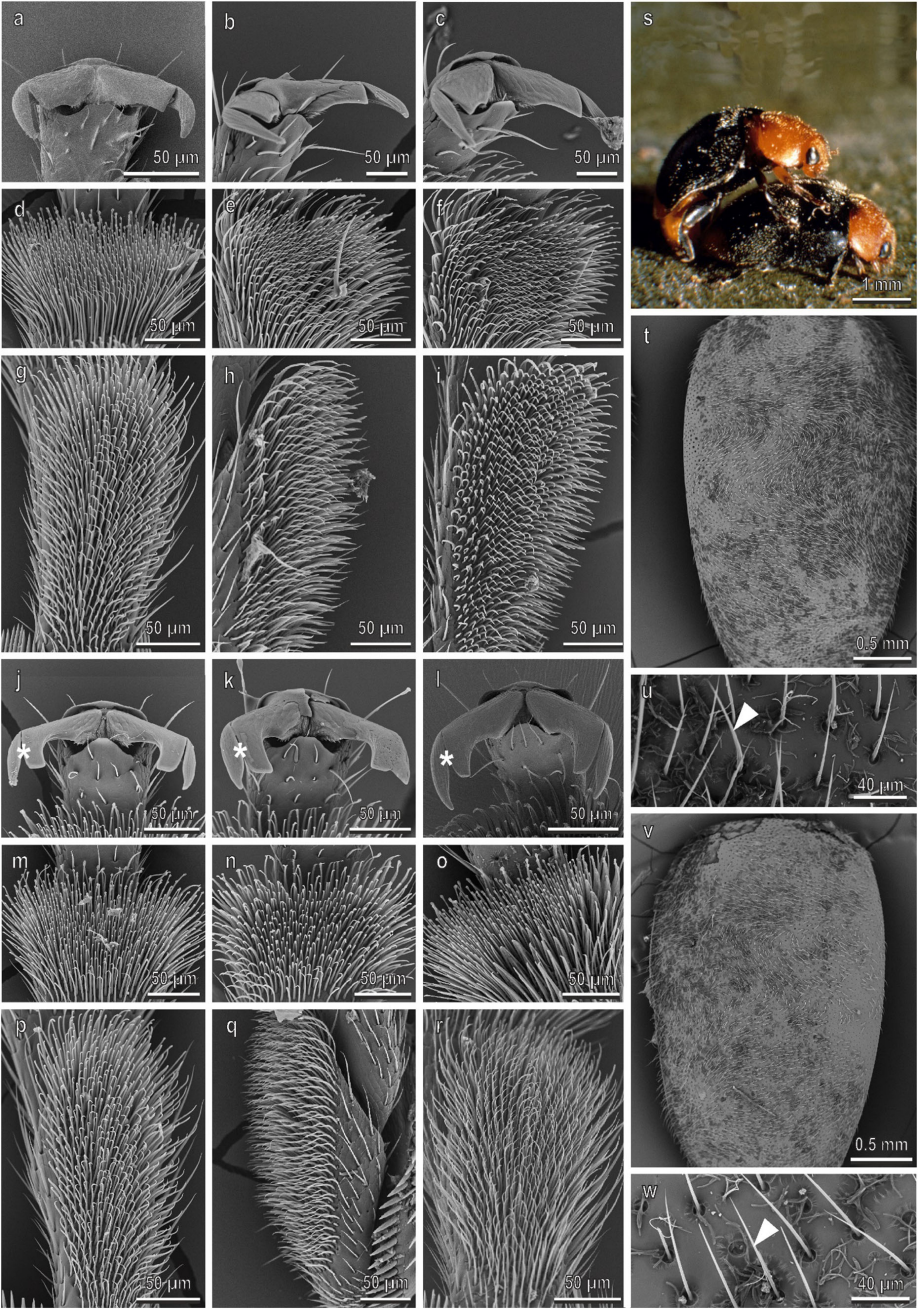
*Cryptoleamus montrouzieri*. Female attachment devices (a–i), female elytra (t and u), male attachment devices (j–r), and male elytra (v and w) in the SEM: claws (a–c and j–l), distal (d–f and m–o) and proximal (g–i and p–r) tarsomeres of the fore‐ (a, d, g, j, m, p), mid‐ (b, e, h, k, n, q), and hindlegs (c, f, i, l, o, r). (s) The position of the male on the female during mating is shown. Arrowheads point to the setae on male and female elytra. The dimorphic claws of male show a more elongated tooth (asterisk) and deeper cleft compared with female.

In *C. montrouzieri*, the pretarsal claws are dentate, with a wide basal tooth in the females and are similar in the three legs (Figure 12a–c). There is a sexual dimorphism in the claw morphology of all the three legs, with the male showing a more elongated tooth and deeper cleft compared with female (compare Figure 12a–c with 12j–l). There is no sexual dimorphism in the structure of the hairy pads (no disco‐setae in the male) (Figure 12m–r). Long setae cover the elytra in both sexes (Figure 12t–w); their density on the female elytra is 589 ± 79 mm^−2^. Wax filaments are clearly visible among the setae of the elytra (Figure 2u,w). Water CA on the female elytra is 169.3° ± 1.5°. During mating (Figures 4 and 12s), male contacts the female elytra using the fore‐ and midlegs, its hindlegs are either on the elytra or raised.

### 3.10 *Exochomus quadripustulatus* (Tribe: Chilocorini)

**Figure 13:**
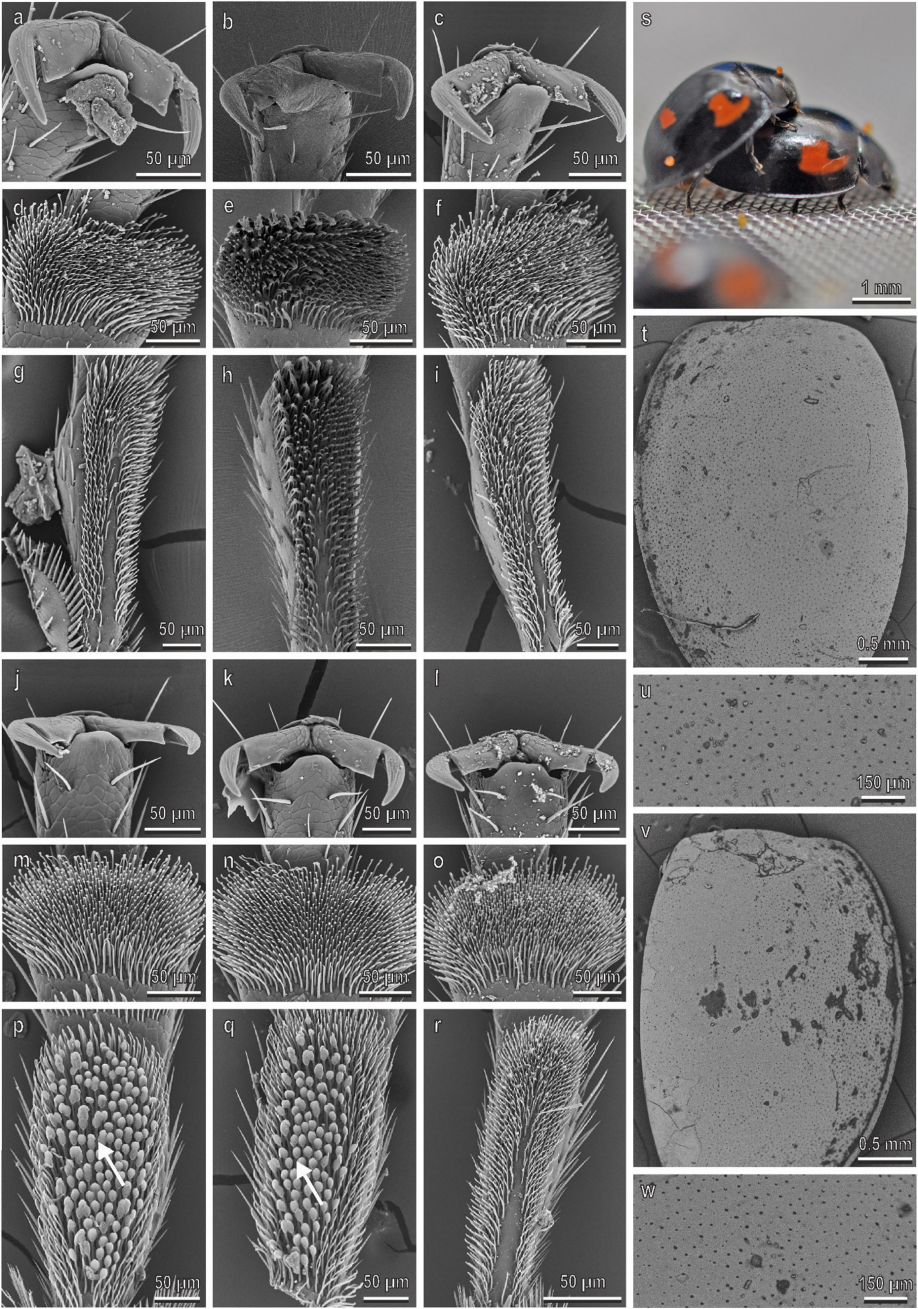
*Exocomus quadripustulatus*. Female attachment devices (a–i), female elytra (t and u), male attachment devices (j–r), and male elytra (v and w) in the SEM: claws (a–c and j–l), distal (d–f and m–o) and proximal (g–i and p–r) tarsomeres of the fore‐ (a, d, g, j, m, p), mid‐ (b, e, h, k, n, q), and hindlegs (c, f, i, l, o, r). (s) The position of the male on the female during mating is shown. Arrows denote the disco‐setae on the male tarsi.

The claws are similar in all the legs of both sexes (Figure 13a–c,j–i) and show a deep cleft separating a wide basal tooth (dentate claws) from the distal portion of the claw. The first tarsomere of the fore‐ and midlegs in the male shows disco‐setae with a wide discoidal terminal plate (Figures 3a and 13p,q). The density of the disco‐setae (about 7000 mm^−2^) is similar (only the first tarsomere has disco‐setae) in both legs (Figure 3a). In both legs, the percentage of the area covered by disco‐setae is about 50% (Figure 3b). The elytra in both sexes are rather smooth, with few scattered pits and short setae (Figure 13t–w). A thin layer of wax covers the elytral cuticle (Figure 13t–w). The female elytra show a water CA of 92° ± 1.6°. During mating, the male is in contact with the female elytra using the fore‐ and midlegs, while the hindlegs are kept on the substrate (Figures 4 and 13s).

### 3.11 *Delphastus catalinae* (Tribe: Seranginini)

**Figure 14:**
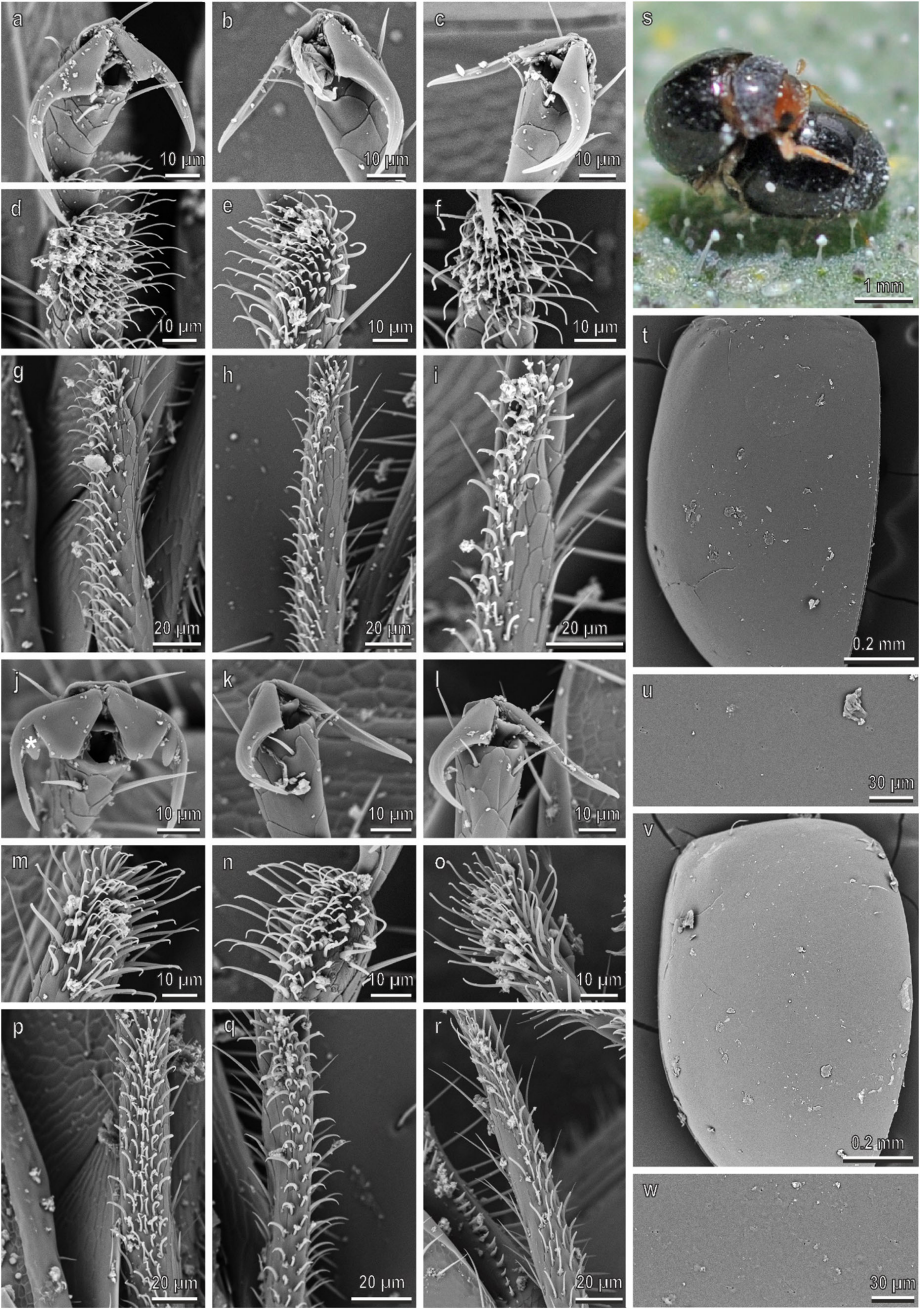
*Delphastus catalinae*. Female attachment devices (a–i), female elytra (t and u), male attachment devices (j–r), and male elytra (v and w) in the SEM: claws (a–c and j–l), distal (d–f and m–o) and proximal (g–i and p–r) tarsomeres of the fore‐ (a, d, g, j, m, p), mid‐ (b, e, h, k, n, q), and hindlegs (c, f, i, l, o, r). In (s) The position of the male on the female during mating is shown. The foreleg of male presents a dimorphic bifid claw (asterisk), not recorded in female.

A pair of simple pretarsal claws similar in the three legs is present in females (Figure 14a–c). There is a sexual dimorphism in the claw morphology of the forelegs, with the male showing bifid dentate claws in the forelegs (compare Figure 14a with 14j), while the claws of mid‐ and hindlegs are similar to those of the female (Figure 14b,c,k,l). No sexual dimorphism is detected in the hairy pads (no disco‐setae in the male pads) (Figure 14m–r). The elytra in both sexes are very smooth, with a thin wax layer covering the cuticle (Figure 14t–w). The female elytra have a water CA of 86.1° ± 1.1°. During mating (Figures 4 and 14s), male interlocks the bifid dentate claws of the forelegs with the anterior edge of the female elytra and keeps its midlegs in contact with the female elytra; its hindlegs are either kept on the elytra or raised.

### 3.12 Overall Comparison

**Figure 15:**
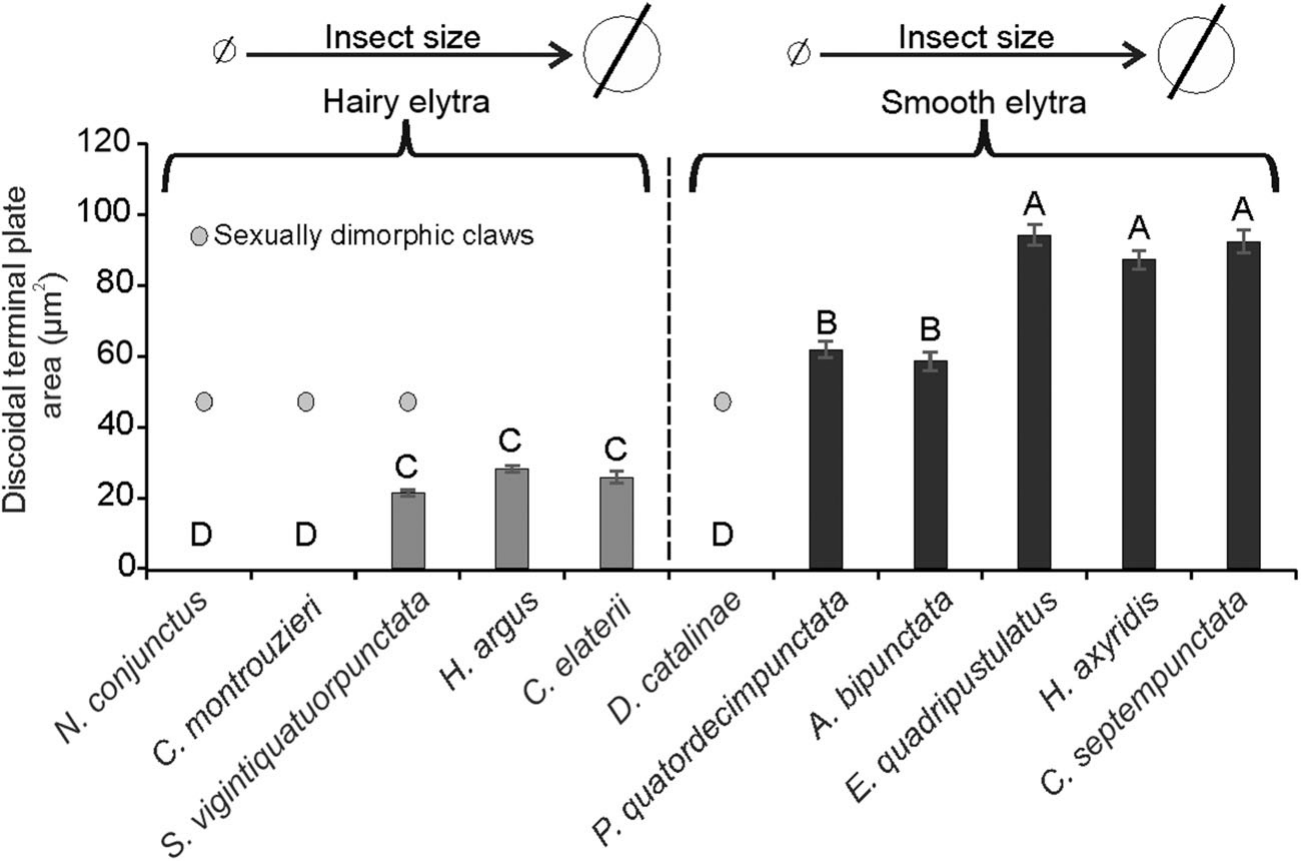
Area (mean ± SE) of discoidal terminal plate of males' specialized disco‐setae and presence of dimorphic claws in relation to the hairy or smooth females' elytra and body size in 11 Coccinellidae species.

On the whole, we can observe that among coccinellid species with hairy elytra, males of species with small sizes (*N. conjunctus* and *C. montrouzieri*) do not possess disco‐setae, but have sexually dimorphic claws, while large‐sized species (*S. vigintiquatuorpunctata*, *H. argus*, and *C. elaterii*) show disco‐setae (Figure 15). The area of discoidal terminal plate of these setae does not significantly differ among species and is smaller in comparison to species with smooth elytra (*F* = 352.1; d.f. = 10, 109; *p* < 0.001) (Figure 15). An intermediate condition is represented by *S. vigintiquatuorpunctata* males, where disco‐setae are present together with slightly modified claws. Among coccinellid species with smooth elytra, small‐sized species (*D. catalinae*) does not show disco‐setae in males, but sexually dimorphic claws, while species of larger sizes have disco‐setae (Figure 15). In the latter cases, the area of discoidal terminal plate is higher in larger species (*E. quadripustulatus*, *H. axyridis*, *C. septempunctata*) and lower in smaller species (*P. quatuordecimpunctata*, *A. bipunctata*) (Figure 15).

## 4 Discussion

In Coleoptera, the structure of female elytra and male attachment devices are shaped by ongoing adaptations and counter‐adaptations driven by reproductive strategies and female mate selection, reflecting a complex interplay of cooperative and noncooperative interactions with males (Alcock 2006). Previous studies have provided evidence of coevolution—sometimes cooperative, sometimes antagonistic—between male attachment organs and female elytra in some selected species from the families of Dytiscidae (Drotz et al. 2010), Chrysomelidae (Voigt et al. 2008), and Coccinellidae (Gorb and Gorb 2020). However, none of them has investigated these aspects in a comparative manner within one family. The present study sheds light for the first time on this relationship in 11 species of Coccinellidae belonging to different tribes from morphological and behavioral perspectives.

Our data reveal that disco‐setae are present only in males of some species and are located on the hairy pads of legs, which hold on female elytra during mating, while these setae are not recorded in legs, which rest on the substrate (Figure 4). Similar situation was found also in the males of the chrysomelid beetle *L. decemlineata*, where the hindlegs lacking disco‐setae are in contact with the female elytra margin and the abdominal sclerites during copulation (Voigt et al. 2008). This phenomenon likely occurs because the evolutionary cost of the sexual dimorphism is justified only for the fore‐ and midlegs that play a greater role in copulation. Ladybirds mate often and for a long time, with duration ranging from 45 min in *C. septempunctata* (Rana and Kakker 2000) to 2–3 h in *H. axyridis* (Hodek et al. 2012) and several days in *Aiolocaria hexaspilota* (Crotch) (Iwata 1932). Such a long mating time requires males to have a strong adhesion system to ensure a successful female insemination. In this context, many authors have emphasized the crucial role of dimorphic setae in Coleoptera during mating (Voigt et al. 2008, 2017; Bullock and Federle 2009; Matsumura et al. 2023). Previous studies comparing the adhesion of disco‐setae with the adhesion of pointed setae on the females body during copulation (Voigt et al. 2008; Matsumura et al. 2023), revealed that the disco‐setae adhere stronger to female surfaces than pointed setae. Matsumura et al. (2023) suggested that the development of these disco‐setae in males could be related to the epicuticular grease layer present on the insect cuticle which makes adhesion particularly challenging. Indeed, disco‐setae are particularly effective on smooth flat or convex surfaces like elytra of some species (Bullock and Federle 2011; Voigt et al. 2017). In rare cases, elytra may be more or less convex. This excellent adhesive property has inspired biologists and engineers to develop bio‐inspired, glue‐free adhesive materials for robotic applications by mimicking the microstructure of beetle and fly feet (Gorb, Sinha et al. 2007).

Elytral setae, along with longitudinal rows and punctures, are a common modification of coleopteran elytral surface (Beutel and Leschen 2016). For example, in many representatives of Dytiscidae, elytra showing a sexual dimorphism are impressed with primary and secondary reticulation in females. These reticulation patterns have taxonomical significance (Drotz et al. 2010) and in the past, were assumed to aid males during mating (Darwin 1871). Further studies suggested that these elytra sculptures reduce male adhesion and are therefore a sexually antagonistic trait associated with sexual conflict (Miller 2003; Karlsson Green et al. 2013; Bilton et al. 2016). To our best knowledge, the function of setae, covering the elytra in Coccinellidae, remains unclear. We hypothesize that for large coccinellids inhabiting dry environments and feeding on plants rich in glandular trichomes (e.g., *C. elaterii* and *H. argus*), the presence of hairy elytra (as well as wax filaments) may help to prevent water loss, as observed in the abdominal cuticle of the tenebrionid beetle *Ulomoides dermestoides* (Fairmaire) (Coleoptera: Tenebrionidae) (Qian et al. 2016). Additionally, our data show that these hairs together with wax filaments render the cuticle to a superhydrophobic state, a property that may: (1) protect phytophagous ladybirds from the sticky plant exudates or glandular trichome secretions; (2) serve as self‐cleaning mechanism against solid particles; (3) shield ladybirds from honeydew produced by prey, such as mealybugs. Further investigations are needed to clarify these aspects.

While comparing the discoidal terminal plate area of male disco‐setae between studied coccinellid species with smooth or hairy elytra (Figure 15), we observed larger terminal elements in species, where females have smooth elytra, and smaller setal tips, when females have hairy elytra. This reduction in size of discoid setal tips is probably an adaptation of male setae for adhesion enhancement due to reaching the smooth surface beneath the elytral setae. Additionally, our investigation revealed that coccinellid species with hairy elytra tend to develop bifid claws (Figures 4 and 15), which may enhance attachment efficiency compared to dentate claws typical in species with smooth elytra. As previously hypothesized by Gorb and Gorb (2020) for *C. montrouzieri*, we can suppose that in species with hairy elytra, males may use their bifid claws with deep clefts to interlock with the elytra setae on the female's elytra during mating. This hypothesis is further supported by the presence of sexual dimorphism in the claw shape found in beetles with hairy elytra, such as *S. vigintiquatuorpunctata*, *N. conjunctus*, and *C. montrouzieri* (Figure 15).

Claws with multiple clefts have been observed in various insect species, which often apply serrate or pectinate claws to attach to surfaces covered by long hair‐like structures. Such claws have been described from the avian ectoparasite *Craterina pallida* Latreille (Diptera: Hippoboscidae), which attaches to the bird feathers using tridentate claws (Petersen et al. 2018), or the bee louse *Braula coeca* Nitzsch (Diptera: Braulidae) attaching to honey bee hairs with its tiny comb‐like claws (Büscher et al. 2021). Similarly, coccinellids with clefts in their claws revealed a higher attachment ability to flexible plant trichomes compared to Coleoptera with simple divergent claws (Salerno, Rebora, Piersanti, Saitta et al. 2022).

In our study, *D. catalinae* was the only species, where we observed a combination of males having bifid claws and smooth female elytra. Here, males use their specialized claws to interlock with the anterior edge of the elytra, a unique coccinellid mating strategy observed only in this small species. Notably, despite the smooth elytra, *D. catalinae* lacks disco‐setae (Figure 4). This finding is particularly intriguing given the small size of beetle individuals. Indeed, the morphology of hairy adhesive organs may be affected also by the animal size (Arzt et al. 2003; Labonte and Federle 2015). It is widely recognized that smaller animals tend to have a better attachment due to their larger surface‐to‐mass ratio compared to larger animals (Labonte and Federle 2015; Salerno et al. 2020). During the ontogenesis, hemimetabolan insects tend to compensate for the decrease of attachment efficiency, caused by a nonproportional increase of the body mass, by enlarging the attachment devices or developing additional adaptations that enhance the attachment performances (Gorb and Gorb 2004; Labonte and Federle 2015) as studied in *Nezara viridula* (L.) (Hemiptera: Pentatomidae) (Salerno et al. 2020) and *Coreus marginatus* (L.) (Hemiptera: Pentatomidae) (Gorb and Gorb 2004). Our data suggest a relationship between the size of coccinellid species (Table S1) and the morphology of male attachment devices used during mating (Figure 15). In particular, we observed that in larger species (ranging from 4 to 8 mm), such as *C. elaterii, H. argus*, *C. septempunctata, H. axyridis*, *A. bipunctata, E. quadripustulatus*, and *P. quatuordecimpunctata*, sexual dimorphism is observed only in adhesive pads (disco‐setae in males) and does not affect the claws. Conversely in smaller species (ranging from 1.5 to 4 mm), such as *C. montrouzieri*, *N. conjunctus*, and *D. catalinae*, sexual dimorphism is present only in claws and does not affect the setae (Figures 4 and 15). It appears that the sexual dimorphism in pad setae, due to its evolutionary cost, develops only in larger species (where no claw dimorphism is observed), whereas in smaller species, claws dimorphism alone is sufficient to ensure a firm hold on the females, avoiding any investment in other adhesive devices. Also in smaller species due to their relatively stronger efficiency of adhesive pads due to the scaling effects mentioned above, the performance of their adhesive pads might be sufficient to maintain strong attachment during copulation even on the challenging surfaces of female elaytra. Interestingly, *S. vigintiquatuorpunctata* stands out as an exception, exhibiting sexual dimorphism in both pad setae and claws. This is probably owed to the intermediate size of this ladybird. We observed that larger coccinellid species, such as *E*. *quadripustulatus*, *C. elaterii*, *H. argus*, and *C. septempunctata*, use occasionally claws to firmly hold on elytra edge. When males interlock their claws with the elytral edge, a significant increase in attachment force can be achieved, as suggested by Voigt et al. (2017) for *C. americana* (Chrysomelidae). In this context, an intriguing structure—the costal edge of the elytra—was reported in *C. septempunctata* by Fu et al. (2024) and it is likely that male claws are adapted to interlock with this elytral rib.

At the end, we would like to emphasize that understanding the fundamental mechanisms that regulate the insect biological cycle (Chown and Nicolson 2004; Shields 2017) could be potentially used for their monitoring and control (Bernays and Chapman 2007; Gorb 2001; Salerno et al. 2021; Schoonhoven et al. 2005).

## 5 Conclusions and Outlook

The present comparative study on 11 species of Coccinellidae belonging to different tribes highlights the co‐evolutionary relationships between the morphology of female elytra and the male attachment devices from the morphological and behavioral points of view. In particular, we show the following: (1) Disco‐setae are present only in males of some species and are located on the hairy pads of legs, which during mating hold on female elytra. These special setae are absent in legs, which are positioned on the substrate. (2) In species, where males have disco‐setae the area of the discoid setal tip is considerably extended, when females have smooth elytra, and is reduced, when females have hairy elytra. (3) Males of coccinellid species with hairy elytra tend to possess bifid claws, which can be useful for mating compared to dentate claws typical in species with smooth elytra. (4) Sexual dimorphism in pad setae evolved only in larger species, where claw dimorphism is absent. In smaller species, the claws dimorphism is sufficient evolutionary adaptation in males, to firmly hold the females, thus avoiding further investment in other attachment devices. These relationships are important for elucidating the evolutionary mechanisms driving diversification within the Coccinellidae family. Furthermore, insights into the functional significance of these morphological traits involved in Coccinellidae mating behavior can provide valuable implications also for pest management strategies, biological control practices (improving the mass rearing techniques), and environmental conservation efforts in this important family of Coleoptera.

## Author Contributions

**Valerio Saitta:** conceptualization, investigation, writing – original draft, writing – review and editing, methodology, data curation. **Manuela Rebora:** conceptualization, writing – original draft, writing – review and editing, methodology, supervision. **Silvana Piersanti:** writing – review and editing. **Giorgia Carboni Marri:** investigation. **Paolo Masini:** visualization. **Elena Gorb:** conceptualization, writing – original draft, writing – review and editing, supervision. **Alessia Iacovone:** investigation, methodology, writing – review and editing. **Gianandrea Salerno:** conceptualization, writing – original draft, writing – review and editing, methodology, formal analysis, data curation, supervision. **Stanislav Gorb:** conceptualization, methodology, writing – review and editing, supervision.

## Supporting information

Supporting information.

## Data Availability

The data that support the findings of this study are available on request from the corresponding author. The data are not publicly available due to privacy or ethical restrictions.

## References

[jmor70041-bib-0001] Ahmad, D. M. E. , D. Nawal , M. Kumari , and K. Kumari . 2024. “Prey and Host Records of *Coccinella* Spp. (Coleoptera: Coccinellidae) in India (A Review).” Journal of Advanced Zoology 45, no. 5: 170–187.

[jmor70041-bib-0002] Alcock, J. 2006. Animal Behavior: An Evolutionary Approach 8th ed. Elsevier.

[jmor70041-bib-0003] Arzt, E. , S. Gorb , and R. Spolenak . 2003. “From Micro to Nano Contacts in Biological Attachment Devices.” Proceedings of the National Academy of Sciences 100, no. 19: 10603–10606. 10.1073/pnas.1534701100 PMC196850 12960386

[jmor70041-bib-0004] Bergsten, J. , A. Töyrä , and A. N. Nilsson . 2001. “Intraspecific Variation and Intersexual Correlation in Secondary Sexual Characters of Three Diving Beetles (Coleoptera: Dytiscidae).” Biological Journal of the Linnean Society 73, no. 2: 221–232.

[jmor70041-bib-0005] Bernays, E. A. , and R. F. Chapman . 2007. Host‐Plant Selection by Phytophagous Insects Vol. 2 Chapman and Hall.

[jmor70041-bib-0006] Beutel, R. G. , and S. N. Gorb . 2001. “Ultrastructure of Attachment Specializations of Hexapods (Arthropoda): Evolutionary Patterns Inferred From a Revised Ordinal Phylogeny.” Journal of Zoological Systematics and Evolutionary Research 39, no. 4: 177–207.

[jmor70041-bib-0007] Beutel, R. G. , and A. B. Leschen . 2016. *Handbook of Zoology: Arthropoda: Insecta. Part. 38. Coleoptera, Beetles*.*Volume 1: Morphology and Systematics (Archostemata, Adephaga, Myxophaga, Polyphaga partim)* 2nd ed. Berlin: Walter De Gruyter.

[jmor70041-bib-0008] Bilton, D. T. , J. W. G. Hayward , J. Rocha , and G. N. Foster . 2016. “Sexual Dimorphism and Sexual Conflict in the Diving Beetle *Agabus uliginosus* (L.) (Coleoptera: Dytiscidae).” Biological Journal of the Linnean Society 119, no. 4: 1089–1095.

[jmor70041-bib-0009] Bullock, J. M. R. , and W. Federle . 2009. “Division of Labour and Sex Differences Between Fibrillar, Tarsal Adhesive Pads in Beetles: Effective Elastic Modulus and Attachment Performance.” Journal of Experimental Biology 212, no. 12: 1876–1888. 19483006 10.1242/jeb.030551

[jmor70041-bib-0010] Bullock, J. M. R. , and W. Federle . 2011. “The Effect of Surface Roughness on Claw and Adhesive Hair Performance in the Dock Beetle *Gastrophysa viridula* .” Insect Science 18, no. 3: 298–304. 10.1111/j.1744-7917.2010.01369.x.

[jmor70041-bib-0011] Büscher, T. H. , D. S. Petersen , N. N. Bijma , et al. 2021. “The Exceptional Attachment Ability of the Ectoparasitic Bee Louse *Braula coeca* (Diptera, Braulidae) on the Honeybee.” Physiological Entomology 47, no. 2: 83–95.

[jmor70041-bib-0012] Canepari, C. 2009. “New Data on Some Coccinellidae (Coleoptera) From the Mediterranean Region.” Zootaxa 2318, no. 1: 394–399.

[jmor70041-bib-0013] Che, L. , P. Zhang , S. Deng , et al. 2021. “New Insights Into the Phylogeny and Evolution of Lady Beetles (Coleoptera: Coccinellidae) by Extensive Sampling of Genes and Species.” Molecular Phylogenetics and Evolution 156: 107045. 33352317 10.1016/j.ympev.2020.107045

[jmor70041-bib-0014] Chown, S. , and S. Nicolson . 2004. Insect Physiological Ecology: Mechanisms and Patterns. Oxford University Press.

[jmor70041-bib-0015] Dai, Z. , S. N. Gorb , and U. Schwarz . 2002. “Roughness‐Dependent Friction Force of the Tarsal Claw System in the Beetle *Pachnoda marginata* (Coleoptera, Scarabaeidae).” Journal of Experimental Biology 205, no. 16: 2479–2488. 10.1242/JEB.205.16.2479. 12124371

[jmor70041-bib-0016] Darwin, C. 1871. The Descent of Man and Selection in Relation to Sex. London: John Murray.

[jmor70041-bib-0017] Dirks, J. H. , and W. Federle . 2011. “Fluid‐Based Adhesion in Insects ‐ Principles and Challenges.” Soft Matter 7, no. 23: 11047–11053. 10.1039/c1sm06269g. 41776880

[jmor70041-bib-0018] Drotz, M. K. , T. Brodin , and A. N. Nilsson . 2010. “Multiple Origins of Elytral Reticulation Modifications in the West Palearctic *Agabus bipustulatus* Complex (Coleoptera, Dytiscidae).” PLoS One 5, no. 2: e9034. 20140264 10.1371/journal.pone.0009034 PMC2815794

[jmor70041-bib-0019] Federle, W. 2004. “Wet Adhesion and Rubber Friction in Adhesive Pads of Insects.” Journal of Adhesion and Interface 5, no. 2: 31–42.

[jmor70041-bib-0020] Frazier, J. L. , and S. Chyb . 1995. “Use of feeding inhibitors in insect control.” In Regulatory Mechanisms in Insect Feeding, edited by R. Chapman , 364–381. Springer US. 10.1007/978-1-4615-1775-7_13.

[jmor70041-bib-0021] Friedemann, K. , R. Spangenberg , K. Yoshizawa , and R. G. Beutel . 2014. “Evolution of Attachment Structures in the Highly Diverse Acercaria (Hexapoda).” Cladistics 30, no. 2: 170–201. 10.1111/cla.12030. 34781597

[jmor70041-bib-0022] Fu, K. , J. Zhang , J. Hu , J. Wu , and Y. Yang . 2024. “Morphological and Structural Characteristics of the Elytra Reduce Impact Damage to Ladybird Beetles.” Journal of Insect Physiology 154: 104630. 10.1016/j.jinsphys.2024.104630. 38432606

[jmor70041-bib-0023] Goczał, J. , and R. G. Beutel . 2023. “Beetle Elytra: Evolution, Modifications and Biological Functions.” Biology Letters 19, no. 3: 202200559. 10.1098/rsbl.2022.0559 PMC9975656 36855857

[jmor70041-bib-0024] Gorb, S. N. 2001. Attachment Devices of Insect Cuticle. Springer Science & Business Media.

[jmor70041-bib-0025] Gorb, S. N. 2005. “Uncovering Insect Stickiness: Structure and Properties of Hairy Attachment Devices.” American Entomologist 51, no. 1: 31–35.

[jmor70041-bib-0026] Gorb, S. N. 2008. “Biological Attachment Devices: Exploring Nature's Diversity for Biomimetics.” Philosophical Transactions of the Royal Society A: Mathematical, Physical and Engineering Sciences 366, no. 1870: 1557–1574. 10.1098/rsta.2007.2172 18192171

[jmor70041-bib-0027] Gorb, S. N. , and E. V. Gorb . 2004. “Ontogenesis of the Attachment Ability in the Bug *Coreus marginatus* (Heteroptera, Insecta).” Journal of Experimental Biology 207, no. 17: 2917–2924. 15277547 10.1242/jeb.01127

[jmor70041-bib-0028] Gorb, E. V. , and S. N. Gorb . 2020. “Attachment Ability of Females and Males of the Ladybird Beetle *Cryptolaemus montrouzieri* to Different Artificial Surfaces.” Journal of Insect Physiology 121: 104011. 10.1016/j.jinsphys.2019.104011. 31904387

[jmor70041-bib-0029] Gorb, E. V. , P. Hofmann , A. E. Filippov , and S. N. Gorb . 2017. “Oil Adsorption Ability of Three‐Dimensional Epicuticular Wax Coverages in Plants.” Scientific Reports 7, no. 1: 1–11. https://www.nature.com/articles/srep45483. 28367985 10.1038/srep45483 PMC5377368

[jmor70041-bib-0030] Gorb, E. V. , N. Hosoda , C. Miksch , and S. N. Gorb . 2010. “Slippery Pores: Anti‐Adhesive Effect of Nanoporous Substrates on the Beetle Attachment System.” Journal of the Royal Society Interface 7, no. 52: 1571–1579. 10.1098/RSIF.2010.0081. 20427333 PMC2988254

[jmor70041-bib-0031] Gorb, E. V. , W. Lemke , and S. N. Gorb . 2019. “Porous Substrate Affects a Subsequent Attachment Ability of the Beetle *Harmonia axyridis* (Coleoptera, Coccinellidae).” Journal of the Royal Society Interface 16, no. 150: 20180696. 10.1098/RSIF.2018.0696. 30958175 PMC6364653

[jmor70041-bib-0032] Gorb, S. N. , M. Sinha , A. Peressadko , K. A. Daltorio , and R. D. Quinn . 2007. “Insects Did It First: A Micropatterned Adhesive Tape for Robotic Applications.” Bioinspiration & Biomimetics 2, no. 4: S117–S125. 10.1088/1748-3182/2/4/S01. 18037721

[jmor70041-bib-0033] Gorb, S. N. , M. Varenberg , A. Peressadko , and J. Tuma . 2007. “Biomimetic Mushroom‐Shaped Fibrillar Adhesive Microstructure.” Journal of the Royal Society Interface 4, no. 13: 271–275. 10.1098/RSIF.2006.0164. 17251156 PMC2359835

[jmor70041-bib-0034] Gorb, E. , D. Voigt , S. D. Eigenbrode , and S. Gorb . 2008. “Attachment Force of the Beetle *Cryptolaemus montrouzieri* (Coleoptera, Coccinellidae) on Leaflet Surfaces of Mutants of the Pea *Pisum sativum* (Fabaceae) With Regular and Reduced Wax Coverage.” Arthropod‐Plant Interactions 2, no. 4: 247–259. 10.1007/S11829-008-9049-0/FIGURES/7.

[jmor70041-bib-0035] Gordon, R. D. 1985. “The Coccinellidae (Coleoptera) of America North of Mexico.” Journal of the New York Entomological Society 93, no. 1: 1–912. https://www.cabdirect.org/cabdirect/abstract/19850528000.

[jmor70041-bib-0036] Heepe, L. , D. S. Petersen , L. Tölle , J. O. Wolff , and S. N. Gorb . 2017. “Sexual Dimorphism in the Attachment Ability of the Ladybird Beetle *Coccinella septempunctata* on Soft Substrates.” Applied Physics A 123, no. 1: 34. 10.1007/S00339-016-0684-5.

[jmor70041-bib-0037] Hodek, I. 1973. Biology of Coccinellidae. Springer Science & Business Media.

[jmor70041-bib-0038] Hodek, I. , A. Honek , and H. V. Emden . 2012. Ecology and Behaviour of the Ladybird Beetles (Coccinellidae). John Wiley & Sons. (ed.).

[jmor70041-bib-0039] Iperti, G. , and P. Prudent . 1986. “Effect of the Substrate Properties on the Choice of Oviposition Sites by *Adalia bipunctata* .” In Ecology of Aphidophaga, edited by I. Hodek , 143–149. Dr. W. Junk Publishers.

[jmor70041-bib-0040] Iwata, K. 1932. “On the Biology of Two Large Lady‐Birds in Japan.” Transaction of the Kansai Entomological Society 3: 13–26.

[jmor70041-bib-0041] Karlsson Green, K. , A. Kovalev , E. I. Svensson , and S. N. Gorb . 2013. “Male Clasping Ability, Female Polymorphism and Sexual Conflict: Fine‐Scale Elytral Morphology as a Sexually Antagonistic Adaptation in Female Diving Beetles.” Journal of the Royal Society Interface 10, no. 86: 20130409. 23825114 10.1098/rsif.2013.0409 PMC3730688

[jmor70041-bib-0042] Labonte, D. , and W. Federle . 2015. “Scaling and Biomechanics of Surface Attachment In Climbing Animals.” Philosophical Transactions of the Royal Society, B: Biological Sciences 370, no. 1661: 20140027. 10.1098/rstb.2014.0027 PMC4275900 25533088

[jmor70041-bib-0043] Liu, Z. , and A. P. Liang . 2016. “Ultramorphology of the Tarsal Adhesive Structures of Eight Leaf Beetle Species (Coleoptera: Chrysomelidae).” Journal of the Kansas Entomological Society 89, no. 3: 215–230.

[jmor70041-bib-0044] Matsumura, Y. , E. V. Gorb , and S. N. Gorb . 2023. “The Tight Attachment Achieved by the Male Discoidal Setae Is Possibly a Counter‐Adaptation to the Grease Layer on Female Integument Surfaces in Green Dock Beetles.” Journal of the Royal Society Interface 20, no. 205: 20230324. 37582406 10.1098/rsif.2023.0324 PMC10427193

[jmor70041-bib-0045] Michels, J. , and S. N. Gorb . 2012. “Detailed Three‐Dimensional Visualization of Resilin in the Exoskeleton of Arthropods Using Confocal Laser Scanning Microscopy.” Journal of Microscopy 245, no. 1: 1–16. 10.1111/j.1365-2818.2011.03523.x. 22142031

[jmor70041-bib-0046] Miller, K. B. 2003. “The Phylogeny of Diving Beetles (Coleoptera: Dytiscidae) and the Evolution of Sexual Conflict: Diving Beetle Phylogeny and Sexual Conflict.” Biological Journal of the Linnean Society 79, no. 3: 359–388.

[jmor70041-bib-0047] Moon, M. J. , H. J. Kim , H. Kim , and J. G. Park . 2012. “Microstructure of the Biological Attachment Devices in the Ladybug *Harmonia axyridis* (Coleoptera: Coccinellidae).” Animal Cells and Systems 16, no. 6: 479–487. 10.1080/19768354.2012.699003.

[jmor70041-bib-0048] Peisker, H. , J. Michels , and S. N. Gorb . 2013. “Evidence for a Material Gradient in the Adhesive Tarsal Setae of the Ladybird Beetle *Coccinella septempunctata* .” Nature Communications 4, no. 1: 1661. 10.1038/ncomms2576 23552076

[jmor70041-bib-0049] Pell, J. K. , J. Baverstock , H. E. Roy , R. L. Ware , and M. E. N. Majerus . 2008. “Intraguild Predation Involving *Harmonia axyridis*: A Review of Current Knowledge and Future Perspectives.” BioControl 53, no. 1: 147–168. 10.1007/S10526-007-9125-X/METRICS.

[jmor70041-bib-0050] Petersen, D. S. , N. Kreuter , L. Heepe , et al. 2018. “Holding Tight to Feathers – Structural Specializations and Attachment Properties of the Avian Ectoparasite *Crataerina pallida* (Diptera, Hippoboscidae).” Journal of Experimental Biology 221, no. 13: jeb179242. 10.1242/jeb.179242. 29712747

[jmor70041-bib-0051] Piersanti, S. , V. Saitta , M. Rebora , and S. Gianandrea . 2023. “Adult Host Preference and Larval Performance in an Ologophagous Insect (*Chnootriba elaterii*).” Physiological Entomology 18, no. 4: 637–649.

[jmor70041-bib-0052] Piersanti, S. , V. Saitta , M. Rebora , and G. Salerno . 2022. “Olfaction in Phytophagous Ladybird Beetles: Antennal Sensilla and Sensitivity to Volatiles From Host Plants in *Chnootriba elaterii* .” Arthropod‐Plant Interactions 16, no. 6: 617–630. 10.1007/s11829-022-09923-y.

[jmor70041-bib-0053] Qian, J. , D. Chi , and R. Chai . 2016. “Possible Functions of the Microtrichia on the Cuticle of *Ulomoides dermestoides* (Chevrolat) (Coleoptera: Tenebrionidae).” Journal of Forestry Research 27: 1391–1405.

[jmor70041-bib-0054] Rana, J. S. , and J. Kakker . 2000. “Biological Studies on 7‐Spot Ladybird Beetle, *Coccinella septempunctata* L. With Cereal Aphid, *Sitobion avenae* (F.) as Prey.” Cereal Research Communications 28: 449–454.

[jmor70041-bib-0055] Rebora, M. , J. Michels , G. Salerno , L. Heepe , E. Gorb , and S. Gorb . 2018. “Tarsal Attachment Devices of the Southern Green Stink Bug *Nezara viridula* (Heteroptera: Pentatomidae).” Journal of Morphology 279, no. 5: 660–672. 10.1002/jmor.20801. 29464747

[jmor70041-bib-0056] Rebora, M. , G. Salerno , S. Piersanti , E. V. Gorb , and S. N. Gorb . 2021. “Attachment Devices and the Tarsal Gland of the Bug *Coreus marginatus* (Hemiptera: Coreidae).” Zoomorphology 140, no. 1: 85–102. 10.1007/S00435-020-00515-Z.

[jmor70041-bib-0057] Rebora, M. , G. Salerno , S. Piersanti , V. Saitta , E. Gorb , and S. N. Gorb . 2022. “Mechanical Interaction of the Egg Parasitoid *Anastatus bifasciatus* (Hymenoptera: Eupelmidae) With Artificial Substrates and Its Host Egg.” Frontiers in Mechanical Engineering 8: 966429. 10.3389/fmech.2022.966429.

[jmor70041-bib-0058] Roy, H. , and E. Wajnberg . 2008. “From Biological Control to Invasion: The Ladybird *Harmonia axyridis* as a Model Species.” BioControl 53, no. 1: 1–4. 10.1007/S10526-007-9127-8.

[jmor70041-bib-0059] Ryan, M. F. , and O. Byrne . 1988. “Plant‐Insect Coevolution and Inhibition of Acetylcholinesterase.” Journal of Chemical Ecology 14, no. 10: 1965–1975. 10.1007/BF01013489. 24277106

[jmor70041-bib-0060] Saitta, V. , M. Rebora , S. Piersanti , E. Gorb , S. Gorb , and G. Salerno . 2022. “Effect of Leaf Trichomes in Different Species of Cucurbitaceae on Attachment Ability of the Melon Ladybird Beetle *Chnootriba elaterii* .” Insects 13, no. 12: 1123. https://www.mdpi.com/2075-4450/13/12/1123. 36555032 10.3390/insects13121123 PMC9787368

[jmor70041-bib-0061] Saitta, V. , M. Rebora , S. Piersanti , E. V. Gorb , G. Salerno , and S. N. Gorb . 2025. “Contribution of Individual Legs to Overall Attachment in the Adult Ladybirds *Harmonia axyridis* Depends on the Relative Leg Orientation to an External Force.” Entomologia Experimentalis et Applicata, Submitted 173, no. 3: 232–245.

[jmor70041-bib-0062] Saitta, V. , M. Rebora , S. Piersanti , and G. Salerno . 2023. “Visual and Chemical Cues in the Host Plant Selection of the Melon Ladybird *Chnootriba elaterii* (Coleoptera: Coccinellidae).” Arthropod‐Plant Interactions 18, no. 4: 637–649.

[jmor70041-bib-0063] Salerno, G. , M. Rebora , S. Piersanti , T. H. Büscher , E. V. Gorb , and S. N. Gorb . 2022. “Oviposition Site Selection and Attachment Ability of *Propylea quatuordecimpunctata* and *Harmonia axyridis* From the Egg to the Adult Stage.” Physiological Entomology 47, no. 1: 20–37. 10.1111/phen.12368.

[jmor70041-bib-0064] Salerno, G. , M. Rebora , S. Piersanti , E. Gorb , and S. Gorb . 2024. “Parasitoid Attachment Ability and the Host Surface Wettability.” Zoology 165: 126181. 38833995 10.1016/j.zool.2024.126181

[jmor70041-bib-0065] Salerno, G. , M. Rebora , S. Piersanti , Y. Matsumura , E. Gorb , and S. Gorb . 2020. “Variation of Attachment Ability of *Nezara viridula* (Hemiptera: Pentatomidae) During Nymphal Development and Adult Aging.” Journal of Insect Physiology 127: 104117. https://www.sciencedirect.com/science/article/pii/S0022191020302614. 33002513 10.1016/j.jinsphys.2020.104117

[jmor70041-bib-0066] Salerno, G. , M. Rebora , S. Piersanti , V. Saitta , E. V. Gorb , and S. N. Gorb . 2022. “Coleoptera Claws and Trichome Interlocking.” Journal of Comparative Physiology, A: Neuroethology, Sensory, Neural, and Behavioral Physiology 209, no. 2: 1–14. 10.1007/S00359-022-01554-1. 35616716 PMC10006029

[jmor70041-bib-0067] Salerno, G. , M. Rebora , S. Piersanti , et al. 2021. “Reduction in Insect Attachment Caused by Different Nanomaterials Used as Particle Films (Kaolin, Zeolite, Calcium Carbonate).” Sustainability 13, no. 15: 8250. 10.3390/SU13158250.

[jmor70041-bib-0068] Schneider, C. A. , W. S. Rasband , and K. W. Eliceiri . 2012. “NIH Image to ImageJ: 25 Years of Image Analysis.” Nature Methods 9, no. 7: 671–675. 10.1002/mrd.22489. 22930834 PMC5554542

[jmor70041-bib-0069] Schoonhoven, L. , J. Van Loon , and M. Dicke . 2005. Insect‐Plant Biology. Oxford University Press.

[jmor70041-bib-0070] Sevarika, M. , and R. Romani . 2024. “Ultrastructural Organization and Metal Elemental Composition of the Mandibles in Two Ladybird Species.” Insects 15, no. 6: 403. 10.3390/insects15060403. 38921118 PMC11203409

[jmor70041-bib-0071] Shields, V. D. , ed. 2017. Biological Control of Pest and Vector Insects. IntechOpen.

[jmor70041-bib-0072] Stork, N. E. 1980. “A Scanning Electron Microscope Study of Tarsal Adhesive Setae in the Coleoptera.” Zoological Journal of the Linnean Society 68, no. 3: 173–306. 10.1111/j.1096-3642.1980.tb01121.x.

[jmor70041-bib-0073] Vantaux, A. , O. Roux , A. Magro , et al. 2010. “Host‐Specific Myrmecophily and Myrmecophagy in the Tropical Coccinellid *Diomus thoracicus* in French Guiana.” Biotropica 42, no. 5: 622–629.

[jmor70041-bib-0074] Voigt, D. , J. M. Schuppert , S. Dattinger , and S. N. Gorb . 2008. “Sexual Dimorphism in the Attachment Ability of the Colorado Potato Beetle *Leptinotarsa decemlineata* (Coleoptera: Chrysomelidae) to Rough Substrates.” Journal of Insect Physiology 54, no. 5: 765–776. 18387627 10.1016/j.jinsphys.2008.02.006

[jmor70041-bib-0075] Voigt, D. , A. Tsipenyuk , and M. Varenberg . 2017. “How Tight Are Beetle Hugs? Attachment in Mating Leaf Beetles.” Royal Society Open Science 4, no. 9: 171108. 28989792 10.1098/rsos.171108 PMC5627132

[jmor70041-bib-0076] Wiegmann, B. M. , and M. D. Trautwein . 2014. “Major Events in the Evolution of Arthropods.” In Princeton Guide to Evolution, 167–173.

[jmor70041-bib-0077] Yao, F.‐L. , S. Lin , L.‐X. Wang , et al. 2021. “Oviposition Preference and Adult Performance of the Whitefly Predator *Serangium japonicum* (Coleoptera: Coccinellidae): Effect of Leaf Microstructure Associated With Ladybeetle Attachment Ability.” Pest Management Science 77, no. 1: 113–125. 10.1002/ps.6042. 32776685

[jmor70041-bib-0078] Younes, G. H. , M. Ahmad , and N. Ali . 2015. “Morphological, Biological and Ecological Studies of the Mycophagous Ladybird *Psyllobora vigintiduopunctata* L. (Coleoptera: Coccinellidae) on Powdery Mildew Fungi in the Coastal Region of Syria.” Jordan Journal of Agricultural Sciences 11, no. 2: 483–494.

