# Supplementary material for: Sexual Dimorphism of Tarsal Attachment Devices and Their Relation to Mating in Coccinellidae
Source: J Morphol. 2025 Apr 3;286(4):e70041. doi: 10.1002/jmor.70041 (PMC11969132; doi:10.1002/jmor.70041)
Supplement: Supplementary file 1 — Supporting information. [file JMOR-286-e70041-s001.docx]

**Supplementary table 1.** Female dimensions (mean ± SE) of 11 species of Coccinellidae with hairy and smooth elytra and their feeding preferences.

| **Coccinellidae species** | **Elytra** | **Body length (mm)** | **Diet** |
| --- | --- | --- | --- |
| ***N. conjunctus*** | Hairy | 1.84 ± 0.03 d | Mealybug |
| ***C. montrouzieri*** | Hairy | 4.46 ± 0.05 c | Mealybug |
| ***S. vigitioquatuorpunctata*** | Hairy | 4.26 ± 0.07 c | Plants from families Caryophillaceae and Fabaceae |
| ***H. argus*** | Hairy | 7.44 ± 0.06 b | *Ecballium elaterium* plants |
| ***C. elaterii*** | Hairy | 7.68 ± 0.07 a | Plants from the family Cucurbitaceae |
| ***D. catalinae*** | Smooth | 1.45 ± 0.03 D | Whiteflies |
| ***P. quatordecimpunctata*** | Smooth | 4.22 ± 0.05 C | Aphids |
| ***A. bipunctata*** | Smooth | 5.27 ± 0.07 B | Aphids |
| ***E. quadripustulatus*** | Smooth | 5.03 ± 0.1 B | Aphids, mealybug |
| ***H. axyridis*** | Smooth | 6.72 ± 0.15 A | Aphids, mealybug and other insects |
| ***C. septempunctata*** | Smooth | 7.04 ± 0.08 A | Aphids and other insects |
